# An Integrated Computational Analysis of High-Risk SNPs in Angiopoietin-like Proteins (ANGPTL3 and ANGPTL8) Reveals Perturbed Protein Dynamics Associated with Cancer

**DOI:** 10.3390/molecules28124648

**Published:** 2023-06-08

**Authors:** Sajid Iqbal, Farida Begum, Dorothy Wavinya Nyamai, Nasir Jalal, Peter Shaw

**Affiliations:** 1 Oujiang Laboratory (Zhejiang Laboratory for Regenerative Medicine, Vision and Brain Health), Wenzhou 325000, China; sajidiqbal@ojlab.ac.cn (S.I.); dorothynyamai@ojlab.ac.cn (D.W.N.); nasirjalal@ojlab.ac.cn (N.J.); 2 Department of Biochemistry, Abdul Wali Khan University Mardan, Mardan 23200, Pakistan; faridaaziz90@yahoo.com; 3 Department of Biochemistry, Jomo Kenyatta University of Agriculture and Technology, Nairobi 00200, Kenya

**Keywords:** angiopoietin-like proteins, high-risk non-synonymous SNPs, protein structure and function, cancer

## Abstract

Angiopoietin-like proteins (ANGPTL) constitute a family of eight proteins (1–8) which play a pivotal role in the regulation of various pathophysiological processes. The current study sought to identify high-risk, “non-synonymous, single-nucleotide polymorphisms” (nsSNPs) in both ANGPTL3 and ANGPTL8 to evaluate the role that these nsSNPs play in various types of cancer. We retrieved a total of 301 nsSNPs from various databases; 79 of these candidates constitute high-risk nsSNPs. Moreover, we identified eleven high-risk nsSNPs that cause various types of cancer: seven candidates for ANGPTL3 (L57H, F295L, L309F, K329M, R332L, S348C, and G409R) and four candidates for ANGPTL8 (P23L, R85W, R138S, and E148D). Protein–protein interaction analysis revealed a strong association of ANGPTL proteins with several tumor-suppressor proteins such as ITGB3, ITGAV, and RASSF5. ‘Gene-expression profiling interactive analysis’ (GEPIA) showed that expression of ANGPTL3 is significantly downregulated in five cancers: sarcoma (SARC); cholangio carcinoma (CHOL); kidney chromophobe carcinoma (KICH); kidney renal clear cell carcinoma (KIRC); and kidney renal papillary cell carcinoma (KIRP). GEPIA also showed that expression of ANGPTL8 remains downregulated in three cancers: CHOL; glioblastoma (GBM); and breast invasive carcinoma (BRCA). Survival rate analysis indicated that both upregulation and downregulation of ANGPTL3 and ANGPTL8 leads to low survival rates in various types of cancer. Overall, the current study revealed that both ANGPTL3 and ANGPTL8 constitute potential prognostic biomarkers for cancer; moreover, nsSNPs in these proteins might lead to the progression of cancer. However, further in vivo investigation will be helpful to validate the role of these proteins in the biology of cancer.

## 1. Introduction

Cancer is the second leading cause of death globally after cardiovascular diseases. Approximately 19.3 million new cases and 10.0 million deaths were reported in 2020 [1]. The global cancer burden is projected to increase to 28.4 million cases by 2040, a 47% increase from 2020, with a larger increase expected in developing (64% to 95%) versus developed (32% to 56%) countries. Notably, this may be further worsened by increasing risk factors associated with globalization and a growing economy [1]. Several studies have been conducted to elucidate the underlying molecular mechanism of pathogenesis to provide information for the development of new and effective strategies to diagnose and treat cancer. Currently, most cancer studies focus on understanding the underlying molecular mechanisms and explore the role of a new class of matricellular proteins [2]. Matricellular proteins are a group of extracellular matrix-associated glycoproteins secreted by cancer cells into the extracellular microenvironment. A recent study demonstrated that these secreted proteins can exacerbate cancer by promoting tumorigenesis [3,4]. ANGPTLs are a family of glycoproteins composed of eight members, abbreviated as ANGPTL1–8 [5]. ANGPTL proteins (1–7) resemble angiopoietins because both families of proteins share two structural domains at both the N-termini and C-termini (although ANGPTL8 constitutes an exception because it lacks a fibrinogen-like structure). Unlike angiopoietins, however, ANGPTLs cannot bind to receptor tyrosine kinases (Tie1 and Tie2) [6]. Hence, this difference suggests that ANGPTLs perform radically different biological functions from similarly structured angiopoietins. ANGPTL proteins have diverse functions and act like endocrine molecules on their corresponding receptor, but their target receptors and underlying functional mechanism(s) still remain largely deciphered [7].

Studies have reported that ANGPTLs are involved in several biological processes, including lipid and glucose metabolisms and redox regulation. All ANGPTL proteins except ANGPTL1, ANGPTL5, and ANGPTL8 promote chronic inflammation. Chronic inflammation is a key factor in cancer cell growth, proliferation, and metastasis [2]. ANGPTL1 inhibits the function of fibroblast growth factor (b-FGF) and vascular endothelial growth factor (VEGF), thus acting as a tumor suppressor [8]. Integrin α1β1 acts as a receptor for ANGPTL1, and their interaction suppresses the phosphorylation of Src and FAK, which, in turn, inhibit the JAK-STAT3 pathway [9]. Previous findings indicate that the high expression of ANGPTL2 in tumor cells can promote metastasis [10]. The expression of ANGPTL3 is significantly upregulated in head and neck squamous cell carcinoma (HNSCC), where it triggers the ERK pathway and promotes cell proliferation [11]. ANGPTL8, also known as lipasin or betatrophin, is a protein encoded by the *C19orf80* gene. This protein is a relatively novel and atypical member of the ANGPTL family; therefore, little is known about its role in diseases. However, its role is implicated in nonalcoholic fatty liver disease (NAFLD) and renal dysfunction [12]. 

The non-synonymous single-nucleotide polymorphisms (nsSNPs) in proteins perturb their structure and function. nsSNPs are single-base changes leading to a change in the amino acid sequence of the encoded protein, and several of these variants are associated with disease [13,14]. These nsSNPs can affect important biological pathways by disrupting protein–protein interaction [15], inducing changes in protein conformation [16], or affecting the post-translational modification sites [17]. As a result, nsSNPs are associated with complex diseases, including cancer. For instance, two SNPs (T266M and E40K) in ANGPTL4 affect triglyceride levels and are associated with cardiovascular disease in type 2 diabetic patients [18]. Similarly, the SNP R59W in ANGPTL8 is associated with low cholesterol levels and also affects the cleavage of ANGPTL3 [19]. Sun et al. reported an nsSNP at position 215 (S215R) in the P53 protein, which is a putative post-translational modification (PTM) site that has been associated with the development of breast cancer [20]. nsSNP mutations in oncoproteins are frequently observed in cancer, and an attempt to restore their functionality is a recent therapeutic strategy. Recent studies revealed that the utilization of in silico approaches for evaluating protein–protein interactions and analyzing SNPs provided evidence that mutations are associated with various disease conditions [13,14,21]. 

**Figure 1:**
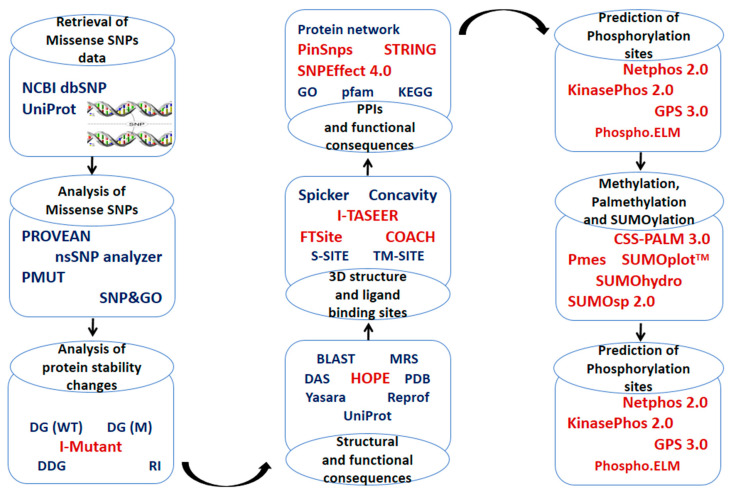
Schematic representation of the workflow used in the current study.

The current study was designed to identify high-risk nsSNPs, SNP-PTM sites, and SNPs located in ligand-binding sites in ANGPTL proteins. Further, protein–protein interaction analysis was conducted and the consequences of SNPs were predicted to investigate the potential role of these proteins in cancer (Figure 1). Our findings indicate that ANGPTL3 and ANGPTL8 play a pivotal role in cancer progression by interacting with tumor-suppressor proteins such as ITGB3, ITGAV, RASSF5, and FNDC5. 

## 2. Results

### 2.1. Missense SNP Datasets

The datasets for ANGPTL3- and ANGPTL8-associated SNPs were retrieved from dbSNP and UniProt databases and were cross checked. We excluded the missing and overlapping data based on flawed sequences. A total of 218 and 83 nsSNPs were retrieved for ANGPTL3 and ANGPTL8, respectively. The SNPs data and their possible effects on protein structure and stability are presented in Supplementary File S1 (Tables S1 and S2). 

### 2.2. Determination of High-Risk Missense SNPs

In the current study, four in silico SNP prediction tools were used, and the SNPs that were predicted as disease-causing by at least three of the predictor’s algorithms were considered high-risk nsSNPs. A total of 67 high-risk SNPs in ANGPTL3 and 12 high-risk SNPs in ANGPTL8 (Supplementary File S1) were identified as high-risk and used for further in-silico analysis (Figure 1). These nsSNPs were analyzed for their effect on protein structure and function using various in silico algorithms, as summarized in Table 1.

### 2.3. Conservation Analysis and Effect of the High-Risk SNPs on Protein Structure Stability

Conservation analysis using the ClustalO tool revealed that all high-risk SNPs in ANGPTL8 were evolutionarily conserved. On the other hand, in ANGPTL3, 50 high-risk missense SNPs were evolutionarily conserved. The protein stability upon a single point mutation was predicted by I-Mutant 3.0 suite. Compared with the wild type, 60 out of 67 SNPs in ANGPTL3 and 10 out of 12 SNPs in ANGPTL8 were predicted to decrease mutant protein stability (Supplementary File S1, Tables S1 and S2).

### 2.4. Prediction of Functional Ligand-Binding Sites

Eight residue sites in ANGPTL3 were predicted to potentially bind with ligands. Further analysis revealed that two high-risk missense SNPs (290 and 409) were located in the ligand-binding site. Four ligand-binding residue sites (32, 35, 36, and 37) in ANGPTL8 were identified by both COACH and FTsite tools. However, no high-risk SNPs were identified in the ligand-binding pocket. 

### 2.5. Prediction of PTM Sites

A total of 49 amino acid residues in ANGPTL3 and 8 residues in ANGPTL8 were phosphorylated. On the other hand, eight O-glycosylation sites were identified in both ANGPTL3 and ANGPTL8. No high-potential ubiquitination sites were predicted in ANGPTL3, whereas the amino acid residue at position 158 was predicted as a potential ubiquitination site in ANGPTL8. Cys residue at position seven was predicted to be a high-potential palmitoylation site in ANGPTL8, whereas no palmitoylation site was predicted in ANGPTL3.

PMes results showed that Arginine at positions 36, 224, 263, 308, 421, 430, and 440 were potential methylation sites in ANGPTL3, whereas no potential methylation site was predicted in ANGPTL8. Lysine residue at positions 5, 34, 97, 105, 114, 131, 139, 165, 219, 416, 419, 423, 425, 435, and 445 was predicted to be acetylated in ANGPTL3. Lysine at positions 62, 124, and 158 was identified as having high potential for acetylation in ANGPTL8.

Amino acid residues at positions 93, 109, and 238 were predicted as having high potential for sumoylation in ANGPTL3. On the other hand, no sumoylation site was identified in ANGPTL8.

### 2.6. High-Risk SNPs Located at PTM and Ligand-Binding Sites

The SNP-PTM overlap analysis revealed that seven SNPs in ANGPTL3 and one high-risk missense SNP in ANGPTL8 overlap with potential PTM sites, but these sites are not highly conserved (Table 2). Moreover, two high-risk SNPs were identified in ANGPTL3 (Supplementary File S2, Figure S1) using COACH and FT Site servers, but no high-risk SNP coincided with ligand-binding residues in ANGPTL8.

### 2.7. ANGPTL 3D Structures, Protein–Protein Interactions, and Molecular Docking

#### 2.7.1. Tertiary Structure and Protein–Protein Interaction Analysis

A total of five models were generated for each protein using the I-TASSER tool, and the model with the highest C-scores was selected for subsequent analysis. The 3D structures of the ANGPTL proteins were visualized using Pymol software. Protein–protein interaction (PPI) analysis revealed that ANGPTL3 interacts with ANGPTL8 (*C19orf80*), LPL, GPIHBP1, ITGB3, ITGAV, GALNT2, PCSK7, TEK, APOA5, and TIE proteins (Figure S2), whereas ANGPTL8 interacts with FNDC5, CUTA, RASSF, INS, ANGPTL4, LPL, ANGPTL3, and GPIHBP1 proteins (Figure S3). The distinct pattern and possible consequences of high-risk SNPs (wild-type and mutant amino acids) on protein structure based on size, charge, and hydrophobicity are shown in Supplementary File S3.

#### 2.7.2. Protein–Protein Docking Using ANGPTL Proteins

**Figure 2:**
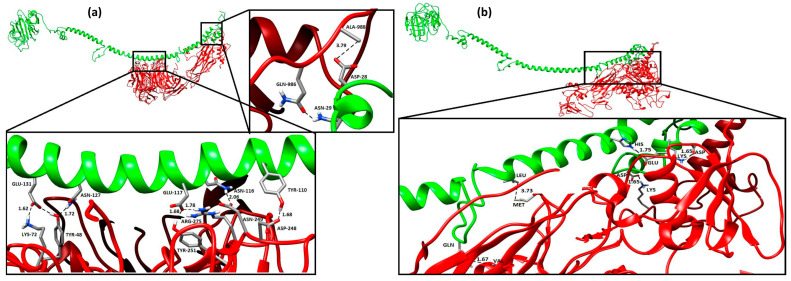
Protein–protein interaction between ANGPTL3-ITGB3 (**a**) and ANGPTL3-ITGAV (**b**).

The protein–protein docking of ANGPTL proteins was performed with the tumor-suppressor proteins, including ITGAV and ITGB3 (data obtained from the STRING database), using the Guru-level interface of the HADDOCK server. HADDOCK clustered 19 structures in two large clusters, representing a 4.7% water-refined model for ANGPTL3 and ITGB3. The top HADDOCK cluster showed that the most reliable structure had a HADDOCK score of −282.8 ± 15.1, Van der Waals energy of −66.2 ± 2.0, electrostatic energy of −226.6 ± 43.3, desolvation energy of −273.6 ± 13, restraint violation energy of 1022.9 ± 58.08, and z-score of −1.0. The topmost cluster structure was refined using the Guru-level interface and further analyzed using the PDBsum server. The PDBsum results showed that 23 interface residues in ANGPTL3 interacted with 23 residues in the ITGB3 protein, including 5 H-bonds, 3 salt bridges, and 117 non-bonded interactions (Figure S4). The interface area (Ȧ°) of the ANGPTL3 protein was 1334 Ȧ°, whereas that of ITGB3 was 1304 Ȧ°. HADDOCK grouped 17 structures in three clusters for ANGPTL3 and ITGAV, presenting 4.25% of the water-refined models. The topmost cluster had a HADDOCK score of −286.6 ± 18.1, Van der Waals energy of −86.6 ± 10.7, electrostatic energy of −182.2 ± 47.4, desolvation energy of −249.8 ± 6.5, restraint violation energy of 863.4 ± 94.56, and z-score of −1.4. The top cluster was refined and subjected to PDBsum analysis. PDBsum results revealed that 23 residues in the ANGPTL3 protein interacted with 28 residues of the ITGAV protein (Figure S4). These interactions comprised 9 H-bonds, 2 salt bridges, and 162 non-bonded interactions. The interface area was 1685 Ȧ° for ANGPTL3 and 1546 Ȧ° for ITGAV. The H-bonds with a distance between the interacting residues lower than 3.5 Ȧ° are shown in Figure 2a,b.

**Figure 3:**
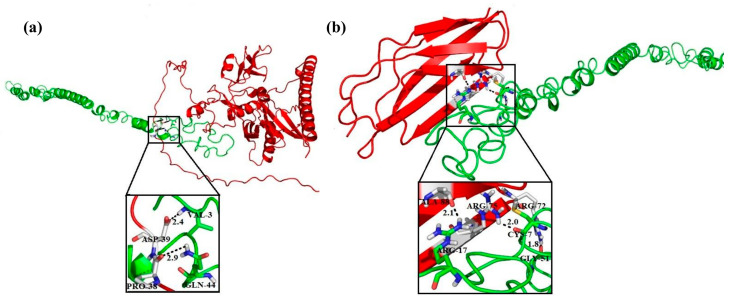
Molecular docking of ANGPTL8 with RASSF5 (**a**) and FNDC5 (**b**).

ANGPTL8 and RASSF5 were docked using HADDOCK, and the most reliable structure had a HADDOCK score of −60.4 ± 15.1. The RMSD of the top structure from the overall lowest energy structure was 1.7 ± 1.7, Van der Waals energy was −85.3 ± 7.5, electrostatic energy was −94.6 ± 27.2, desolvation energy was −23.6 ± 7.5, restraint violation energy was 675.3 ± 153.8, and z-score was −1.6. PDBsum results indicated that 28 interface residues in ANGPTL8 interacted with 24 residues in the RASSF5 protein, and the interactions included 5 H-bonds and 138 non-bonded contacts (Figure S5). The interface area (Ȧ°) of ANGPTL8 was 1363 Ȧ°, whereas that of RASS5 was 1384 Ȧ°. The H-bonds with a distance between the interacting residues lower than 3 Ȧ° are shown in Figure 3a.

ANGPTL8 and FNDC5 were docked using the HADDOCK tool, and 123 structures were clustered in 13 clusters, representing 61% of the water-refined models generated. The statistics of the top 10 clusters were a HADDOCK score of −59.7 ± 6.1, RMSD from the overall lowest energy structure of 15.5 ± 0.1, Van der Waals energy of −53.8 ± 6.3, electrostatic energy of −146.2 ± 26.2, desolvation energy of −11.3 ± 5.2, restraint violation energy of 345.9 ± 41.4, and z-score of −1.8. The PDBsum results showed that 19 interface residues in ANGPTL8 interacted with 12 residues in FNDC5, including eight H-bonds (Figure S5). The interface area of ANGPTL8 was 1334 Ȧ°, whereas that of FNDC5 was 1363 Ȧ°. The H-bonds with a distance between the interacting residues lower than 3 Ȧ° are shown in Figure 3b.

### 2.8. High-Risk nsSNPs Associated with Cancer

Our results indicated that eight SNPs in ANGPTL3 were associated with the development of various types of cancer. The SNPs including L57H, F295L, L309F, K319M, R332L/Q, S384C, and G409R were predicted to cause kidney, lung, endometrial, skin, large intestine, esophageal, and CNS cancer, respectively. In ANGPTL8, four SNPs were associated with cancer. These SNPs were located at positions 23, 85, 138, and 148 and associated with large intestine (P23L and R85W), breast (R138S), and liver (E148D) cancer (Supplementary File S1, Table S3).

### 2.9. Structure Comparison between Wild-Type and Mutated Proteins

The structures of the wild-type and mutated ANGPTL3 and ANGPTL8 proteins were generated using I-TASSER. In both cases, the most suitable model (based on z-score) was selected. Seven high-risk SNPs were incorporated during the modeling of ANGPTL3, and four SNPs were incorporated in the generated 3D structure of the ANGPTL8 protein. We compared the two structures (wild and mutated) using TM-align to determine the structural impact of the high-risk SNPs on the proteins. The RMSD and TM-score of the mutated structure of ANGPTL3 were 3.27 and 0.74, respectively, which indicated that the structures did not exhibit the same conformation and the mutated structure was significantly different from the wild-type protein structure. The high-risk SNPs also perturbed the structure of the ANGPTL8 protein with an RMSD value of 5.38 and a TM-score of 0.45. A TM score of 0.0–0.30 indicates random structural similarity, whereas a score of 0.5–1.00 indicates that the structures are in the same field. The structures of ANGPL3 (wild and mutated) and ANGPTL8 (wild and mutated) are superposed and shown in Figure S6.

### 2.10. Expression Analysis of ANGPTL3 and C19orf80

**Figure 4:**
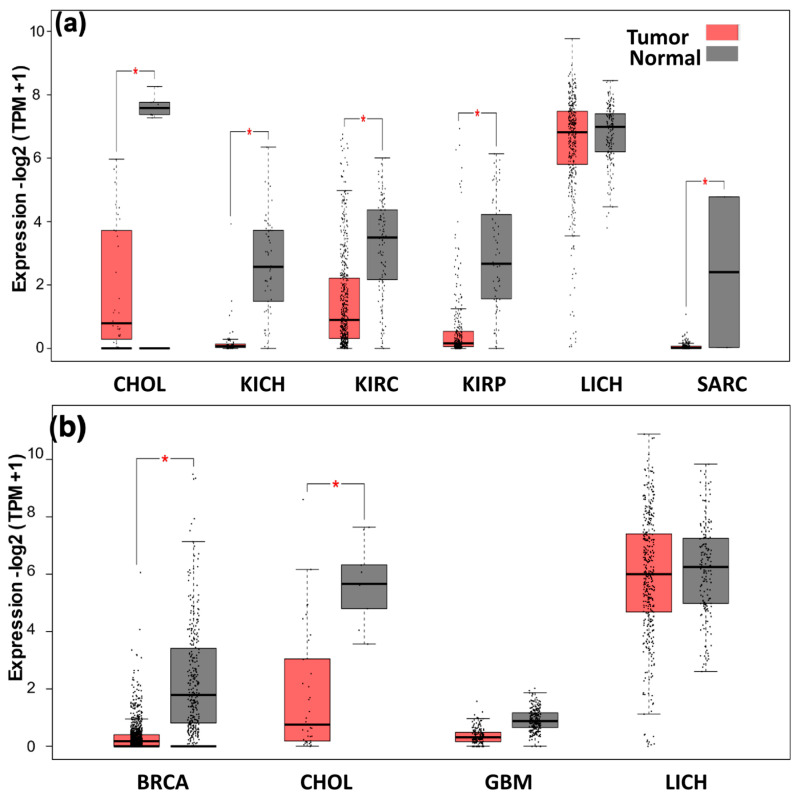
The expression levels (box plot) of *ANGPTL3* (**a**) and *C19orf80* (**b**) in cancer and normal tissues. The box boundaries mark the 25th and 75th percentile, and the line within the box indicates the median. The dots above and below the box mark the 90th and 10th percentiles. The relative expression data were analyzed using the 2 −ΔΔCq method. Asterisk (*) represents significant (*p* < 0.05) differences between tumor and adjacent normal tissue.

The expression levels of the *ANGPTL3* gene in cholangio carcinoma (CHOL), kidney chromophobe (KICH), kidney renal clear cell carcinoma (KIRC), kidney renal papillary cell carcinoma (KIRP), liver hepatocellular carcinoma (LIHC), and sarcoma (SARC) were evaluated, and the results are presented as a box plot. The results showed that *ANGPTL3* expression is significantly downregulated in all these cancers except LIHC (Figure 4a). On the other hand, *C19orf80* expression is downregulated in breast invasive carcinoma (BRCA), CHOL, and glioblastoma (GBM) (Figure 4b). The expression of *C19orf80* is also altered in LIHC but remains insignificant. The protein-coding mRNA levels of the genes in various types of cancer and adjacent normal tissue were analyzed using RNA sequence data to compare the expression level of *ANGPTL3* and *C19orf80* in normal and tumor tissues. A dot plot was generated which revealed that the *ANGPTL3* expression level was downregulated in KICH, KIRC, KIRP, and CHOL. On the other hand, the expression level of *C19orf80* was downregulated in BRCA, CHOL, and BGM (Figure S7).

### 2.11. Survival Analysis in Cancer Patients

**Figure 5:**
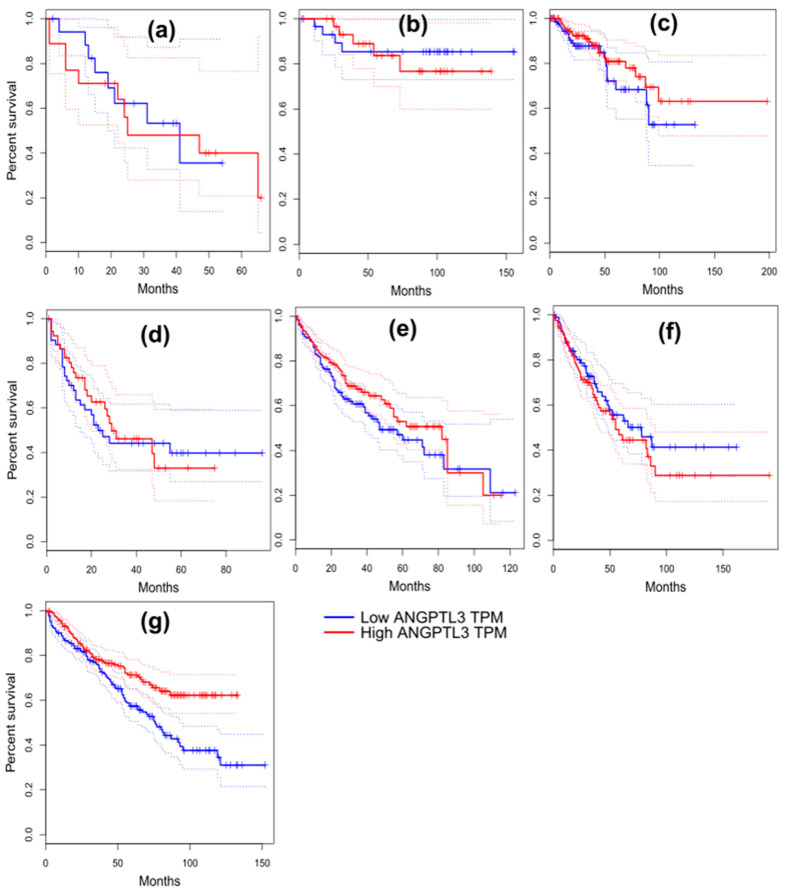
Kaplan–Meier overall survival (%) analysis for ANGPTL3 in patients suffering from cholangio carcinoma (CHOL) (**a**), kidney chromophobe (KICH) (**b**), kidney renal papillary cell carcinoma (KIRP) (**c**), acute myeloid leukemia (LAML) (**d**), liver hepatocellular carcinoma (LIHC) (**e**), sarcoma (SARC) (**f**), and kidney renal clear cell carcinoma (KIRC) (**g**).

**Figure 6:**
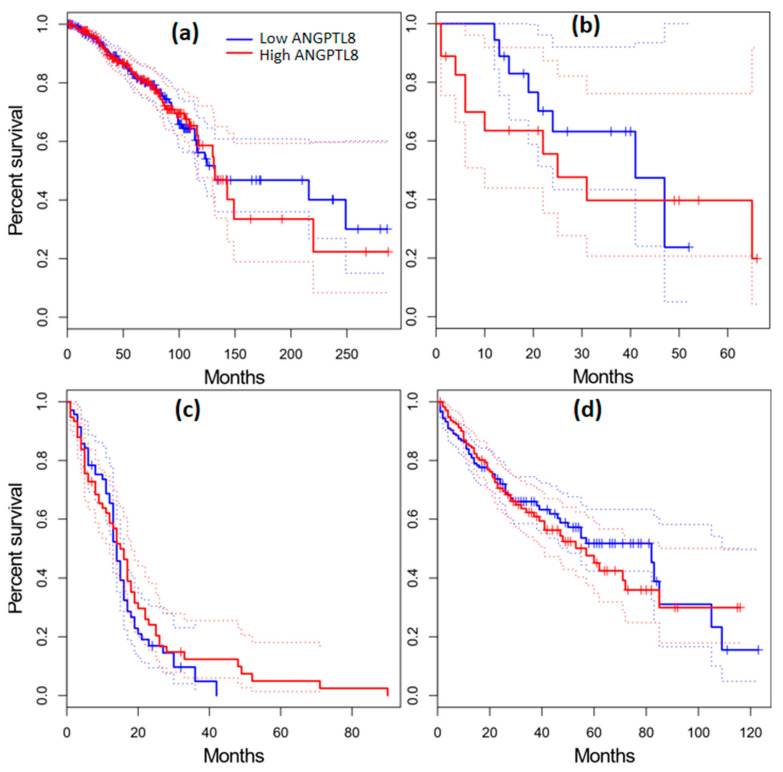
Kaplan–Meier overall survival (%) analysis for ANGPTL8 in BRCA (**a**), CHOL (**b**), GBM (**c**), and LICH (**d**) patients.

The GEPIA database was utilized to evaluate the survival outcome of patients diagnosed with various types of cancer. The patients were grouped into low- and high-expression groups based on the median expression level of ANGPTL3 and ANGPTL8. Dysregulated expression of ANGPTL3 and ANGPTL8 was associated with poor prognosis in cancer patients. For instance, low expression of ANGPTL3 was correlated with a shorter survival time of KIRP and SARC patients (Figure 5c,f). In contrast, a lower expression of ANGPTL3 was associated with a longer survival rate of KICH, LAML, and LIHC cancer patients (Figure 5b,d,e). On the other hand, the overexpression of ANGPTL8 was correlated with significantly longer survival time of CHOL and GBM patients (Figure 6). These results indicate that the dysregulated expression of ANGPTL3 and ANGPTL8 promotes the development of various types of cancer. 

### 2.12. Association between Mutation and Expression Level of ANGPTL3 and C19orf80 with Other Genes

**Figure 7:**
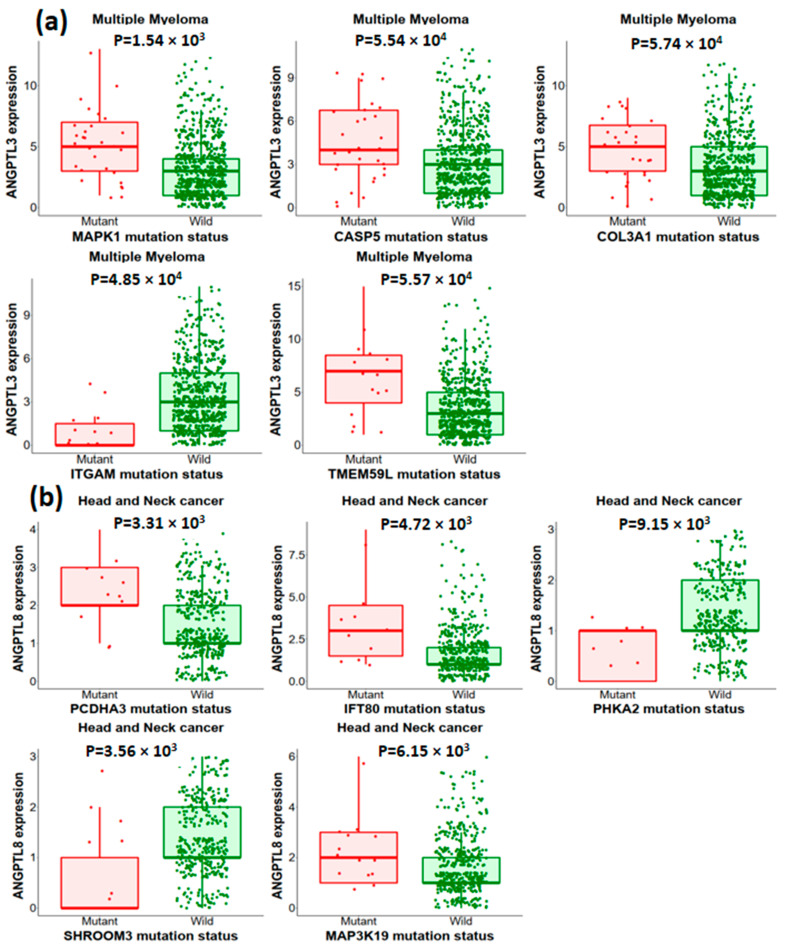
Mutations of other genes are correlated with the expression of ANGPTL3 (**a**) and ANGPTL8 (**b**) in multiple myeloma and head and neck cancer, respectively.

muTarget is an online server for the prediction of the relationship between mutation and alteration in gene expression. The muTarget “Genotype” run analysis revealed that *ANGPTL3* mutation affects the expression of other tumorigenic genes including *LINC02167*, *MYCL*, *TESMIN*, *VCP*, and *TARBP1*. The “Target” run with *ANGPTL3* as a target gene shows that altered genes of various cancer types including *MAPK1*, *CASP5*, *COL3A1*, *ITGAM* and *TMEM59L* change the expression of *ANGPTL3* (Figure 7a). Similarly, the expression of *C19orf80* can be altered due to the mutation in genes associated with various types of cancer such as head and neck cancer and lung squamous cell carcinoma (Figure 7b).

## 3. Discussion

ANGPTL3 and ANGPTL8 are multifunctional secreted proteins commonly expressed in the liver and are regulated by several post-translational modifications (PTM). Previous findings revealed that ANGPTL3 plays a key role in various biological processes such as angiogenesis, lipid metabolism, and tumorigenesis. ANGPTL8 is an atypical member of the angiopoietin-like protein family. This protein is associated with inflammation and metabolic syndromes, and was recently reported to be involved in hepatocellular carcinoma [8]. The alignment of ANGPTL3 and ANGPTL8 sequences exhibited 50% identity at the N-terminal. The structural conformation of a protein plays a critical role in executing its function; however, nsSNPs may perturb the conformations of proteins. Therefore, it is important to explore the effect of pathological nsSNPs on changes in the structure of protein and their association with various disorders. Recent predictions of nsSNPs in oncogenes from large datasets using bioinformatic investigation show their adverse effect on protein structure and function [22]. In the present study, we used integrated computational analysis to determine the high-risk SNPs and their effect on the structure and function of ANGPTL3 and ANGPTL8 proteins. Four prediction tools (nsSNP analyzer, PROVEAN, SNP&Go, and PMUT) were used to explore the pathological effect, and the SNPs predicted as pathological by at least three tools were considered high-risk SNPs. We identified 67 high-risk nsSNPs in ANGPTL3 and 12 nsSNPs in ANGPTL8. In ANGPTL3, seven high-risk SNPs coincided with PTM sites, of which five were identified in highly conserved sites, whereas two SNPs were located in the ligand-binding site. A previous study showed that protein function is upregulated or downregulated by several PTMs, such as the phosphorylation of LC3A, which downregulated its autophagic function, whereas dephosphorylation enhanced its autophagic function [23]. Moreover, another study demonstrated that amino acid residue R68 found in the ligand-binding site in Atg8 is required for interaction with Atg4B [24]. Protein structure, function, and regulation significantly depend on protein stability. A decrease in protein stability causes aggregation, degradation, and misfolding, which leads to the dysfunction of proteins. Protein stability change was determined to evaluate the effect of the predicted high-risk SNPs. Out of the 67 SNPs in ANGPTL3, 60 decreased protein stability and therefore may result in a dysfunctional protein. On the other hand, 10 out of 12 SNPs in ANGPTL8 were predicted to decrease protein stability. 

The evolutionary conservation of a high-risk pathological SNP further increases the severity of a mutation. The SNPs that are located in highly conserved regions are likely to have severe pathological effects compared with those found in the variable regions. In ANGPTL3, out of 67 high-risk SNPs, 50 were located in highly conserved regions, and 5 were identified in PTM sites. A previous study reported a mutation T352 in BECNI1 located in a highly conserved region which modulated the autophagy mechanism and enhanced the interaction between the encoded protein and Bcl-2 [25]. 

NetSurfP offers evolutionary conservative data with solvent accessibility prediction. The tool is used to predict whether a residue is functional (buried) or structural (exposed) based on the position relative to the protein core or surface. In this study, 18 SNPs out of the 67 were identified as functional, whereas 49 were predicted to be structural residues in ANGPTL3. Six out of the twelve SNPs in ANGPTL8 were predicted to be functional residues. These results indicate that ANGPTL3 and ANGPTL8 contain several high-risk SNPs due to their high conservancy, functionality, and ability to decrease protein stability. Protein stability cannot be predicted based on a single factor. The physiochemical properties of residues such as hydrophobicity, charge, and polarity are also important factors in determining protein stability. Protein stability is associated with the presence of hydrophobic amino acids on the protein’s surface area, as these amino acids hinder water access to the core protein area. For instance, among the exposed SNPs, G54R introduces positive charges R (arginine) in place of neutral G (glycine), which causes the repulsion of a ligand or other residues with the same charge. As a result, this mutation may severely hamper the interaction between ANGPTL3 and other molecules. Similarly, the SNP S292P, mutant (proline) residue bigger than the wild-type (serine) residue causes bumps. In addition, the wild-type residue is hydrophobic and forms a H-bond with aspartic acid at position 292, whereas the mutant residue is not involved in H-bond formation and increases the hydrophobicity of the protein. This finding is consistent with a previous study which reported that mutation at position 292 (rs138899888) inhibits protein secretion associated with plasma triglyceride level [26]. In ANGPTL8, the SNP at position 66, serine (S), is substituted with isoleucine (I). The mutation introduces a more hydrophobic residue at this position which results in the loss of hydrogen bonds and might disrupt correct protein folding. These results were verified through a HOPE analysis. The results from the HOPE tool showed that 49 high-risk SNPs in ANGPTL3 and 12 SNPs in ANGPTL8 have a deleterious effect on the protein structures (Supplementary File S3). A PPI network is a critical parameter to understand the cellular processes associated with a protein. STRING is a tool used to evaluate and filter functional proteomic data which offers an intuitive program for determining the structural, functional, and evolutionary features of proteins. The PPI analysis revealed that ANGPTL3 interacts with integrin β3 (ITGB3) and ITGAV. ITGB3 is a cell-adhesion receptor that plays a crucial role in the interaction between cells and extracellular fluid, and it proposed to be critical for cancer metastasis. 

Protein docking analysis showed that 23 interface residues of ANGPTL3 interact with 23 residues of cancer-suppressing ITGB3 and ITGAV proteins. The interactions included H-bonds, salt bridges, and non-bonding interactions. The residue (Leu) at position 57 in ANGPTL3 interacts with the Asp688 of ITGB3 (Figure S4). The mutation at this position (L57H) was identified as a high-risk nsSNP and was predicted to cause kidney cancer. A study involving the Chinese Han population demonstrated that two SNPs (rs11137037 and rs12674822) in ANGPTL2 are associated with the progression of lung cancer [27]. ANGPTL8 was shown to interact with 24 and 12 residues of RASSF5 and FNDC5 proteins, respectively. RASSF5 is involved in a series of cellular processes, such as cell cycle regulation, cell proliferation, and differentiation, and the inactivation of RASSF5 has been implicated in oncogenesis. The overall tertiary structural consequences of these pathological SNPs were further evaluated through Phyre2 homology modeling. The wild-type and mutant protein structures were generated and the TM-align tool was used to determine the RMSD and TM score for the models. A lower TM score and higher RMSD values indicate a higher deviation of the mutant protein structure from the wild type. The TM alignment results revealed that the conformations of ANGPTL3 and ANGPTL8 proteins were significantly different compared with the native protein. The number of cancer-causing high-risk SNPs was lower in ANGPTL8 (*n* = 4) than in ANGPTL3 (*n* = 7). However, more adverse effects were observed on the ANGPTL8 structure than in ANGPTL3 (Figure S6). This can be attributed to the vast physiochemical differences in amino acids between wild-type and mutant proteins. The mutations in protein not only affect protein structure and function but also affect protein expressions, which may lead to disease conditions.

Gene expression analyses indicated that lower expression levels of *ANGPTL3* and *C19orf80* are associated with cancer. A box plot was generated for various types of cancer to show the effect of the downregulation of *ANGPTL3* and *C19orf80* genes more explicitly. The results showed that CHOL, KICH, KIRC, KIRP, LIHC, and SARC cancers were associated with the downregulation of *ANGPTL3* (Figure 5a). On the other hand, BRCA, CHOL, and GBM were associated with the downregulation of *C19orf80* expression (Figure 5b). These results indicate that normal expression of *ANGPTL3* and *C19orf80* is critical for normal function, as the downregulation of these genes causes various types of cancers in humans. 

Median cancer survival time (Kaplan–Meier plot) was estimated by constructing a survival curve that provides a summary of data and survival probability. The survival analysis showed that CHOL, KIRP, and SARC patients with low expression levels of ANGPTL3 had a shorter survival time, whereas KICH, LAML, and LIHC cancer patients with low expression levels of ANGPTL3 exhibited a high survival rate (Figure 5b,d,e). The overexpression of *C19orf80* in CHOL and GBM patients was associated with a significantly longer survival time (Figure 6). These results are consistent with the analysis results obtained for *ANGPTL3* and *C19orf80* expression. These findings indicate that the downregulation and upregulation of ANGPTLs can promote cancer development. The underlying molecular mechanism of this process should be elucidated and may be a hot topic for future investigations. 

The muTarget is a robust tool for predicting target gene expression due to mutation as well as identifying the genes whose mutation affects the expression of another target gene. Our analysis revealed that the expression of various tumorigenic genes, including *LINC02167*, *MYCL*, *TESMIN*, *VCP*, and *TARBP1*, was altered by the mutations in the *ANGPTL3* gene. *MYCL* is a proto-oncogene associated with the apocrine adenosis of breast and lung cancer [28]. The expression of the *TARBP1* gene is upregulated in hepatocellular carcinoma (HCC), and its expression is associated with the pathological severity of the disease [29]. Analysis using “Target run” showed that mutations in *MAPK1*, *CASP5*, *COL3A1*, *ITGAM*, and *TMEM59L* genes changed the expression profile of *ANGPTL3* (Figure 7a). The *MAPK1* encodes a well-known protein (kinase 1) involved in several cell signaling pathways, such as cell differentiation, proliferation, transcriptional regulation, and cellular development [30]. A recent study reported that *ITGAM* regulates myeloid cell polarization and tumor growth. The authors further suggested that *ITGAM* negatively regulates immune suppression; therefore, it is a potential target for cancer immunotherapy [31]. The mutations in various genes, such as *PCDHA3* and *MAP3K19*, altered the expression of *C19orf80* in head and neck cancer patients (Figure 7b). 

The *PCDHA3* gene inhibits epithelial–mesenchymal transition and the metastasis of squamous cell lung carcinoma [32]. Recent findings indicate that *MAP3K19* activates ERK and JNK kinases, therefore enhancing the viability of mutant lung cancer cells [33]. The expression of *ANGPTL3* changed in breast cancer patients owing to the mutations in genes, including *TP53* and *PDZD2*. Intriguingly, several tumor-suppressor genes, i.e., *TP53*, *MAPK1*, and *PDZD2*, altered the expression of target genes, which indicates that mutation in one tumor-suppressor gene may negatively affect other genes of the related category. This relation may further exacerbate the disease condition. *MAPK1* is also involved in transcriptional regulation. This type of gene-to-gene interaction can be used to establish tumor biomarkers as well as therapeutic drug targets. Seven high-risk SNPs in ANGPTL3 and four in ANGPTL8 were predicted to be involved in cancer. This study provides a framework for a new role of ANGPTL3 and ANGPTL8, as well as information on functional mutations. However, in the future, in vivo studies should be conducted to validate the significance of the correlation of these SNPs in ANGPTL3 and ANGPTL8 with a specific type of cancer. 

## 4. Material and Methods

### 4.1. Retrieval of Protein Sequence Datasets and Identification of High-Risk SNPs

The human ANGPTL3 and ANGPTL8 amino acid sequences were retrieved from the UniProt sequence database https://www.uniprot.org/ accessed on 25 February 2023 (Supplementary File S1). The missense SNP data for ANGPTL3 and ANGPTL8 were retrieved from dbSNP [34]. The accession numbers and amino acid sequences of both proteins are provided in Supplementary File S2. To predict functional consequences of missense SNPs, various web-based computational tools including nsSNP analyzer [35], SNP&GO [36], Protein Variation Effect Analyzer (PROVEAN) [37], and PMUT [38] were used. The SNPs were considered high risk when they were predicted as pathological by at least three separate tools. 

### 4.2. Conservation Pattern of High-Risk SNPs in ANGPTL3 and ANGPTL8

Amino acid sequence conservation is associated with protein structure and function. ConSurf predicts the conservation pattern of amino acids in proteins based on the phylogenetic relationships between homologous sequences. The tool uses a color-coded scheme to present conservation scores from low to high (1–9) and classifies amino acids into variable, average, and highly conserved groups. Amino acid sequences of both ANGPTL3 and ABGPTL8 were used as input to ConSurf (https://consurf.tau.ac.il/consurf_index.php) (Accessed on 3 December 2022). Further, the conservation pattern in various species including *Homo sapiens*, *Bos taurus*, *Mus musculus*, and *Rattus norvegicus* was evaluated using the Clustal Omega (https://www.ebi.ac.uk/Tools/msa/clustalo/) (accessed on 6 December 2022) multiple sequence alignment tool.

### 4.3. Prediction of Changes in Protein Stability Induced by High-Risk SNPs in ANGPTL3 and ANGPTL8

I-mutant version 3.0, a web-based support vector machine (SVM) tool, was employed to prioritize the change in protein stability due to high-risk missense SNPs. I-mutant computes Gibbs free energy (DDG) change using the following formula, [39]

Gibbs free energy (DDG) = Mutant protein-free energy − wild-protein free energy

The free energy is calculated in kcal/mol.

The energy change analyses were performed at default parameters with pH 7 and temperature 25 °C. The functionally deleterious or pathological SNPs were distinguished from neutral ones based on the concept that protein stability perturbation must be above a specific free energy threshold of ±1 kcal/mol.

### 4.4. Structural Consequences of High-Risk nsSNPs

The HOPE (Have Our Protein Explained) tool was used to predict the structural consequences of high-risk nsSNPs in ANGPTL proteins. The HOPE tool uses PDB, BLAST, FASTA, HSSP, DSSP, Clustal Omega, YASARA, and DAS web servers to predict the structural effect of SNPs in protein and also constructs independent homology models [40]. PinSnps was utilized to predict the phenotypic consequences of high-risk SNPs in ANGPTL proteins [41]. PinSnps is one of the largest repositories of SNPs data, comprising 3,209,256 SNPs from the dbSNP database with 10,850 disease-related SNPs and 1,101,990 SNPs related to cancer (https://fraternalilab.kcl.ac.uk/PinSnps/) (accessed on 1 April 2023). SNPeffect4.0 was employed to predict changes in integration prediction (TANGO), chaperone-binding prediction (LIMBO), amyloid prediction (WALTZ), and protein stability (DDG) score for a specific missense mutation [42].

### 4.5. Tertiary Structure and Ligand-Binding Site Prediction

The tertiary structures of ANGPTL proteins were not found in the Protein Data Bank (PDB) database. Therefore, the structure of both ANGPTL proteins was predicted using the Iterative Threading ASSEmbly Refinement (I-TASSER) server (https://zhanglab.ccmb.med.umich.edu/I-TASSER/) (accessed on 14 November 2022). The ligand-binding sites were predicted using COACH (http://zhanglab.umich.edu/COACH/) (accessed on 16 November 2022) and FTSite (http://ftsite.bu.edu/) (accessed on 12 January 2022) servers. The residues sites simultaneously predicted by both servers were considered ligand-binding pockets.

### 4.6. Prediction of PTMs Sites

#### 4.6.1. Phosphorylation, Kinase-Specific Phosphorylation, and O-Glycosylation Sites

Phosphorylation is a chemical process that involves the addition or transfer of phosphate (PO43-) group to serine (Ser), Threonine (Thr), or Tyrosine (Tyr), primarily associated with protein activity and protein expression regulation. Netphos 2.0, an artificial-neural-network-based bioinformatics tool, was employed to identify phosphorylation sites in human ANGPTL proteins [43]. The threshold value >0.8 was selected to indicate potential phosphorylation sites. Subsequently, kinase-specific phosphorylation residues were identified using GPS 3.0 [44] and KinasePhos 2.0 [45]. YinOYang, a web-based server [45], was employed to predict O-glycosylation sites in ANGPTL3 and ANGPTL8. NetsurfP [46] was used to determine the surface accessibility of predicted phosphorylation sites.

#### 4.6.2. Ubiquitylation Sites

Three web-based bioinformatics tools, iUbiq-lys (http://www.jci-bioinfo.cn/iUbiq-Lys) (accessed on 24 November 2022), CKSAAP UbSite (http://protein.cau.edu.cn/cksaap_ubsite/) (accessed on 24 November 2022), and BDM-PUB (http://bdmpub.biocuckoo.org/prediction.php) (accessed on 8 November 2022), were employed to identify ubiquitination sites in all ANGPTL proteins. Ubiquitination sites predicted by at least two servers were considered to have a high potential for ubiquitination.

#### 4.6.3. Palmitoylation and Methylation Sites

CSS-PALM 3.0 (http://csspalm.biocuckoo.org/index.php), (accessed on 22 November 2022) a java-based web server, was employed to identify potential palmitoylation sites in ANGPTLs with CC and CXXC patterns. C indicates Cysteine and X indicates any amino acid residue. PMes (http://bioinfo.ncu.edu.cn/inquiries_PMeS.aspx) (accessed on 14 November 2022), a support vector machine program, was used to identify methylation sites in ANGPTL proteins.

#### 4.6.4. Acetylation and Sumoylation Site

PAIL (http://bdmpail.biocuckoo.org/results.php) (accessed on 10 November 2022) and ASEB (http://bioinfo.bjmu.edu.cn/huac/predict_p/) (accessed on 13 November 2022), web servers, were used to predict acetylation sites in ANGPTL proteins. Sumoylation is a reversible modification associated with many diseases. Three online computational tools, SUMOplot^TM^ (http://www.abgent.com/sumoplot) (accessed on 9 December 2022), SUMOhydro (http://protein.cau.edu.cn/) (accessed on 16 November 2022), and SUMOsp 2.0 (http://sumosp.biocuckoo.org/) (accessed on 8 January 2023), were used to predict sumoylation sites in ANGPTL proteins. The threshold for SUMOplot was set at >0.6 and the medium level was selected for prediction using the SUMOsp 2.0 tool. The sumoylation sites predicted by at least 2 servers were considered high-potential sumoylation sites.

### 4.7. Protein–Protein Interactions (PPIs) and Prediction of High-Risk SNPs Associated with Cancer

The STRING (Search Tool for the Retrieval of Interacting Genes/Proteins) database (http://string-db.org/cgi/input) (accessed on 28 November 2022) and PinSnps (http://fraternalilab.kcl.ac.uk/PinSnps/) (accessed on 29 November 2022) were utilized to perform PPI analysis. PinSnps was also used for the prediction of high-risk SNPs associated with various types of cancer. 

### 4.8. Protein Structure Preparation and Docking

All 3D protein structures were assessed using ERRAT score, i.e., amino acids with an ERRAT score confidence level lower than 90%, were rejected [47]. RAMPAGE was utilized to produce Ramachandran plots. Ramachandran plots provide information on the percentage of the individual residue in a specific position [48]. The structure was uploaded to Guru-level HADDOCK (High Ambiguity Driven protein–protein Docking) interface prediction server [49]. The top cluster was further refined for better orientation to improve the HADDOCK score. C-port (consensus prediction of interface residues in transient complexes) was utilized to predict the active and passive residues in ANGPTLs and interacting proteins. The structures were prepared in Chimera and then submitted to C-port to predict active and passive residues [50]. 

### 4.9. Comparative Modeling and Visualization of Wild-Type Proteins and Their Mutated Structures

Predictive 3D modeling was performed along with the structural comparison between wild-type and mutated models to explore whether the high-risk SNPs significantly alter the resultant mutated protein. The nsSNPs at the specific positions in the native protein sequence were made and I-TASSER was used for the structural modeling of mutated ANGPTL3 and ANGPTL8 proteins. The mutated models were then analyzed using the TM-align tool (https://zhanggroup.org/TM-align/) (accessed on 4 April 2023). The TM-align output consists of a modeling score (0–1) and root mean square deviation (RMSD) value for the comparison of protein structures based on the superimposition of structure to determine the differences. 

### 4.10. Analysis of Survival Rate and Gene Expression Analysis

The gene expression profiling interactive analysis (GEPIA) tool presents the expression profiles of a specific gene as a box plot or dot plot and performed overall survival analysis using the log-rank test (http://gepia.cancer-pku.cn/) (accessed on 14 March 2022). GEPIA is an interactive database that is used to interpret RNA sequencing expression data from the genotype tissue expression GTEx project and cancer genome atlas (TCGA) database. The name of the candidate gene, i.e., *ANGPTL3* and *C19orf80* (ANGPTL8) and cancer type were used as input for the expression profile and survival analysis. 

### 4.11. Analysis of the Correlation between Expression Level and Mutation in ANGPTL3 and C190orf80 Genes

The correlation between gene expression and mutations was analyzed using a “genotype” or “target” run. The genotype run determines the changes in gene expression that are related to a specific mutation. The target run is used to predict mutations that affect the expression of a target gene. muTARGET uses the TCGA database to determine the correlation between gene expression and somatic mutation implicated in cancer (https://www.mutarget.com/analysis) (accessed on 13 March 2023).

## 5. Conclusions

The current study’s findings indicate that ANGPTL3 and ANGPTL8 proteins are tumor suppressors and are aberrantly expressed in various types of cancer. Mutations in these proteins are suggested to be associated with perturbed protein structure and function. A total of 79 high-risk nsSNPs were identified in ANGPTL3 and ANGPTL8; these are potentially pathological and decrease protein stability, suggesting their deleterious effect on protein structure. Further analysis showed seven high-risk SNPs (L57H, F295L, L309F, K329M, R332L, S348C, and G409R) in ANGPTL3 and four high-risk SNPs (P23L, R85W, R138S, and E148D) in ANGPTL8 associated with various types of cancer, including large intestine cancer, breast cancer, liver cancer, skin cancer, and head and neck cancer. We believe that the comprehensive analysis of nsSNPs in ANGPTL3 and ANGPTL8 represents a significant advancement in our understanding of these proteins and their role in cancer. By understanding the genetic basis of cancer, researchers and healthcare providers are in a better position to provide personalized cancer screening and prevention strategies. We hope the current study will serve as a benchmark for the development of potential diagnostic tools and therapeutic interventions. However, a large-scale, population-based study should be conducted to validate the effect of these nsSNPs on the structure and function of the proteins and their associations with cancer. 

## Figures and Tables

**Table 1:** Summary of the pathological nsSNPs and their effects.

Purpose	Tool Used	ANGPTL3		ANGPTL8	
Effect of SNP on protein	nsSNP analyzer	Pathological	Neutral	Pathological	Neutral
		83	135	12	71
	SNP&GO	115	103	38	45
	PROVEAN	67	151	30	53
	PMUT	78	140	4	79
Total high-risk SNPs	Predicted by 3 or >3	67		12	
Protein stability upon SNP	I-Mutant	Decrease	Increase	Decrease	Increase
		60	7	6	1
No. of conserved residues	ClustalO	50		All	
Ligand binding	FTsite and COACH	8		0	
Phosphorylation sites	Netphos 2.0	49		8	
Glycosylation sites	YinOYang	8		5	
Ubiquitination sites (≥2)	iUbiq-lys, CKSAAP UbSite, BDM-PUB	0		1	
Palmitoylation sites	CSS-PALM 3.0	0		1	
Methylation sites	PMes	7		0	
Acetylation sites	PAIL and ASEB	15		3	
Sumoylation sites	SUMOplot^TM^ SUMOhydro and SUMOsp	3		0	
Total PTM sites	-	82		18	

**Table 2:** The functional consequences of high-risk missense SNPs in ANGPTL proteins, perturbing PTM sites, ligand-binding sites, and associated cancer types.

Protein	High-Risk SNPs	Conserved (100%)	I-Mutant Analysis			PTM Site	Ligand-Binding Site	Associated Cancer Type
			DDG	Stability	Reliability Index			
ANGPTL3	L57H	Yes	−0.44	Decrease	5	-	-	Kidney
	T64K	Yes	−2.41	Decrease	8	Yes	Yes	-
	T64R	Yes	−1.33	Decrease	3	-	Yes	-
	S292P	Yes	−1.41	Increase	2	Yes	-	-
	F295L	Yes	−2.25	Decrease	6	-	-	Lung
	L309F	Yes	−0.18	Decrease	6	-	-	Endometrium
	K319M	Yes	−0.15	Increase	3	-	-	Skin
	Y321D	Yes	−0.32	Decrease	4	Yes	-	-
	R332L	Yes	−1.64	Decrease	9	-	-	Large intestine
	R332Q	Yes	−1.59	Decrease	9	-	-	Large intestine
	S348C	Yes	−1.27	Decrease	2	Yes	-	Esophagus
	Y358H	Yes	−0.35	Decrease	6	Yes	-	-
	G409R	Yes	0.14	Decrease	5			CNS
	S446P	No	−1.63	Increase	3	Yes	-	-
	S447I	No	−1.69	Decrease	6	Yes	-	-
ANGPTL8	P23L	No	0.06	Increase	3	-	-	Large intestine
	R85W	No	−0.17	Decrease	7	-	-	Large intestine
	R138S	No	−1.18	Decrease	9	-	-	Breast
	E148D	Yes	−0.65	Decrease	7	-	-	Liver

## Data Availability

The protein sequences and nsSNP data used in the current study are freely available from UniProt (https://www.uniprot.org/) and dbSNP (https://www.ncbi.nlm.nih.gov/projects/SNP/) databases, respectively.

## Supplementary Materials

The following supporting information can be downloaded at: https://www.mdpi.com/article/10.3390/molecules28124648/s1, Figure S1: ANGPTL3 3D structure cartoon and sphere representation, Figure S2: Protein-protein interaction analysis network of ANGPTL3 with other related proteins., Figure S3: Protein-protein interaction analysis network of ANGPTL8 with other related proteins, Figure S4: Interaction of ANGPTL3 (Chain A) with ITGB3 (Left) and ITGAV (right), Figure S5: Interaction of ANGPTL8 (Chain A) with RASSF5 (Left) and FNDC5 (right), Figure S6: Comparative homology modeling of wild type and mutated ANGPTL3 and ANGPTL8. The wilt-type structures are shown in green while mutants are shown in red color, Figure S7: Gene expression analysis of ANGPTL3 and C19orf80 in all tumor types paired with normal tissue using the GEPIA database; Table S1: Prediction results for nsSNPs in the ANGPTL3 protein, Table S2: Prediction results for nsSNPs in the ANGPTL8 protein, Table S3: Details of high-risk SNPs in ANGPTL3 and ANGPTL8 that cause cancer.

## Sample Availability

Samples of the compounds are not available from the authors.
