# Supplementary material for: An Integrated Computational Analysis of High-Risk SNPs in Angiopoietin-like Proteins (ANGPTL3 and ANGPTL8) Reveals Perturbed Protein Dynamics Associated with Cancer
Source: Molecules. 2023 Jun 8;28(12):4648. doi: 10.3390/molecules28124648 (PMC10303706; doi:10.3390/molecules28124648)
Supplement: Supplementary file 1 [file molecules-28-04648-s001.zip › Supplementary file S2.pdf]

ANGPTL proteins entry name and accession no.

ANGPTL3 are Q9Y5C1 (Human), Q2KJB3 (Bovine), Q9R182 (Mouse), F7FHP0 (Rat), and for ANGPTL8; Q6UXH0 (Human), E1BKI5 (Bovine), Q8R1L8 (Mouse), D3ZMI0 (Rat)

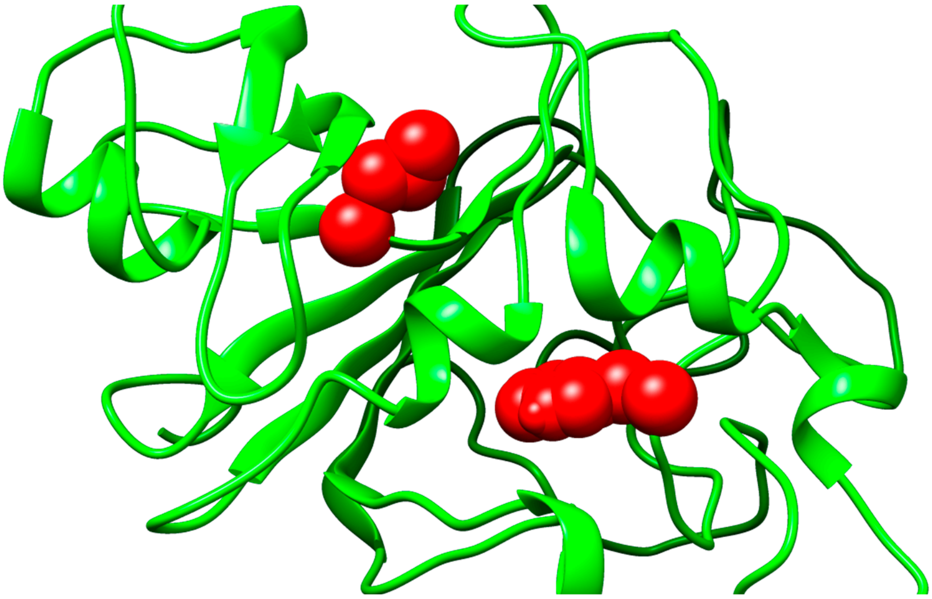

Figure S1. ANGPTL3 3D structure cartoon and sphere representation; 3D structure representing high-risk SNPs (Leu290 and Trp409) lying in ligand binding pockets predicted by COACH and FTSite server together.

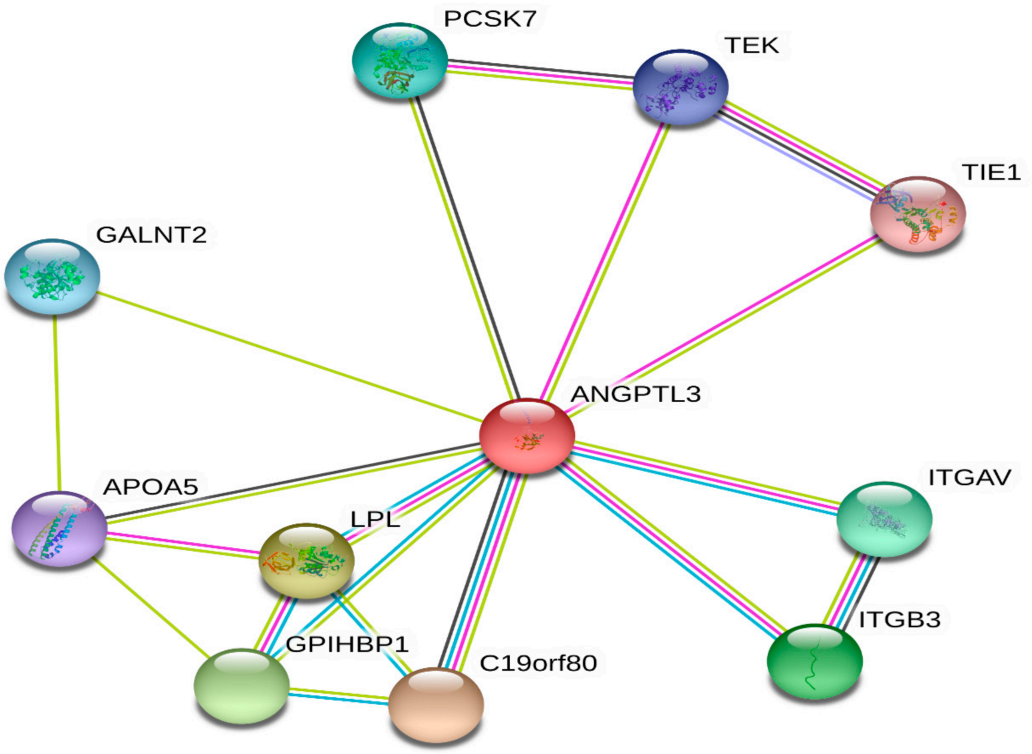

Figure S2. Protein-protein interaction analysis network of ANGPTL3 with other related proteins.

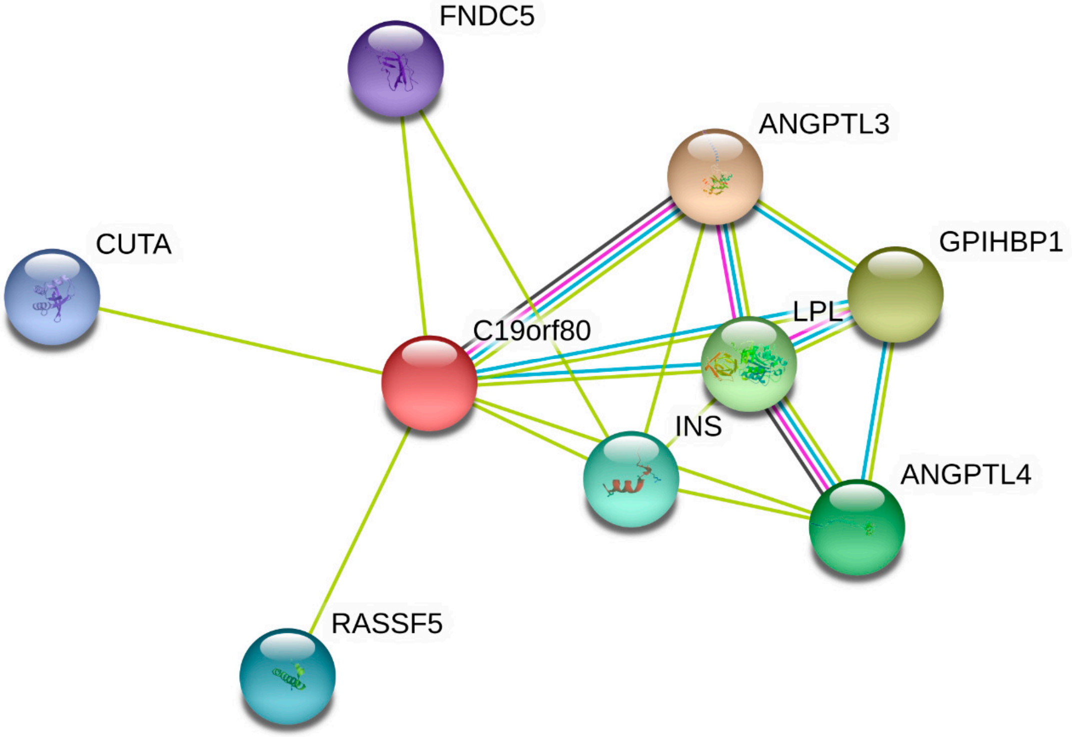

Figure S3. Protein-protein interaction analysis network of ANGPTL8 with other related proteins.

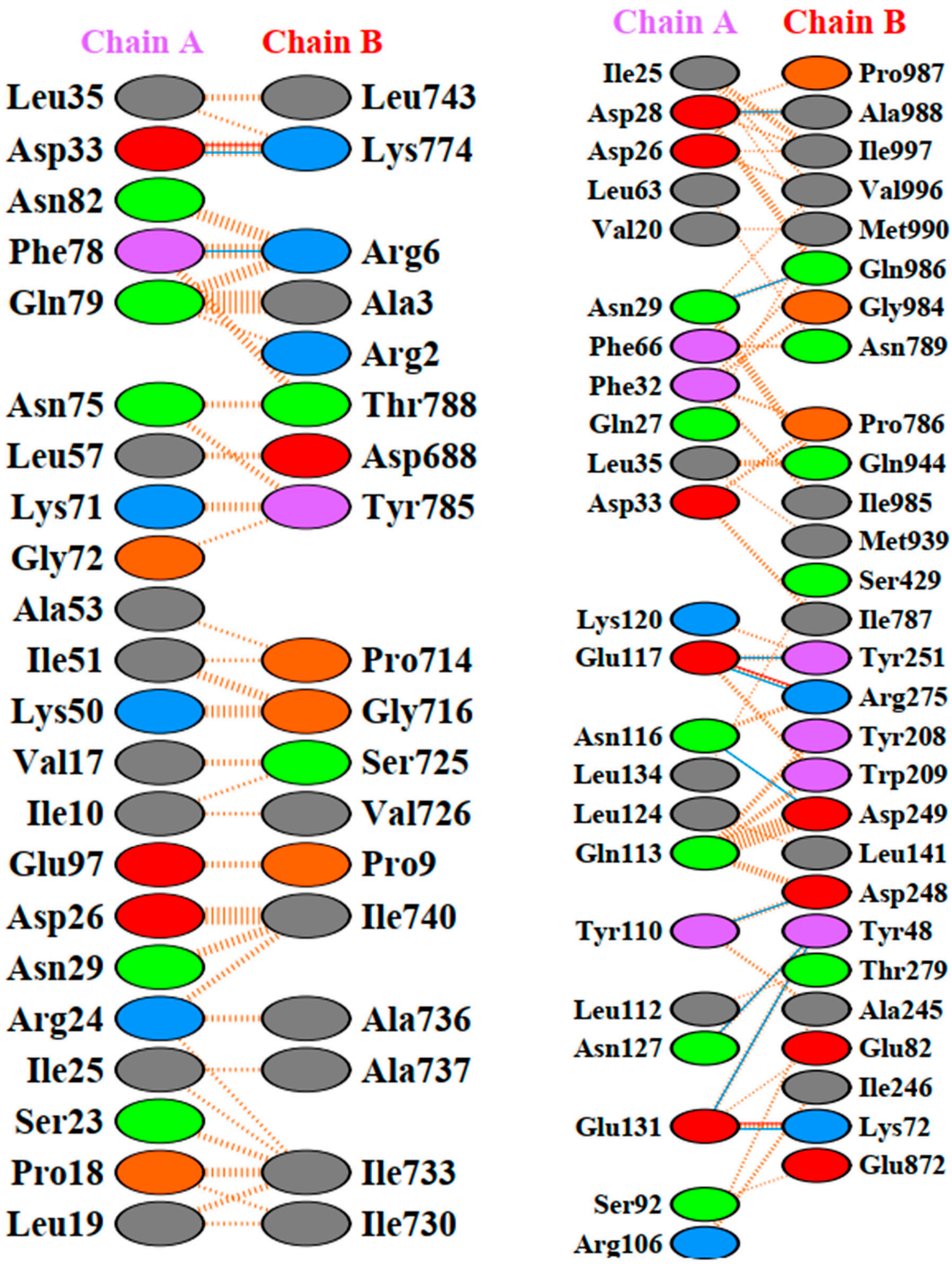

Figure S4. Interaction of ANGPTL3 (Chain A) with ITGB3 (Left) and ITGAV (right).

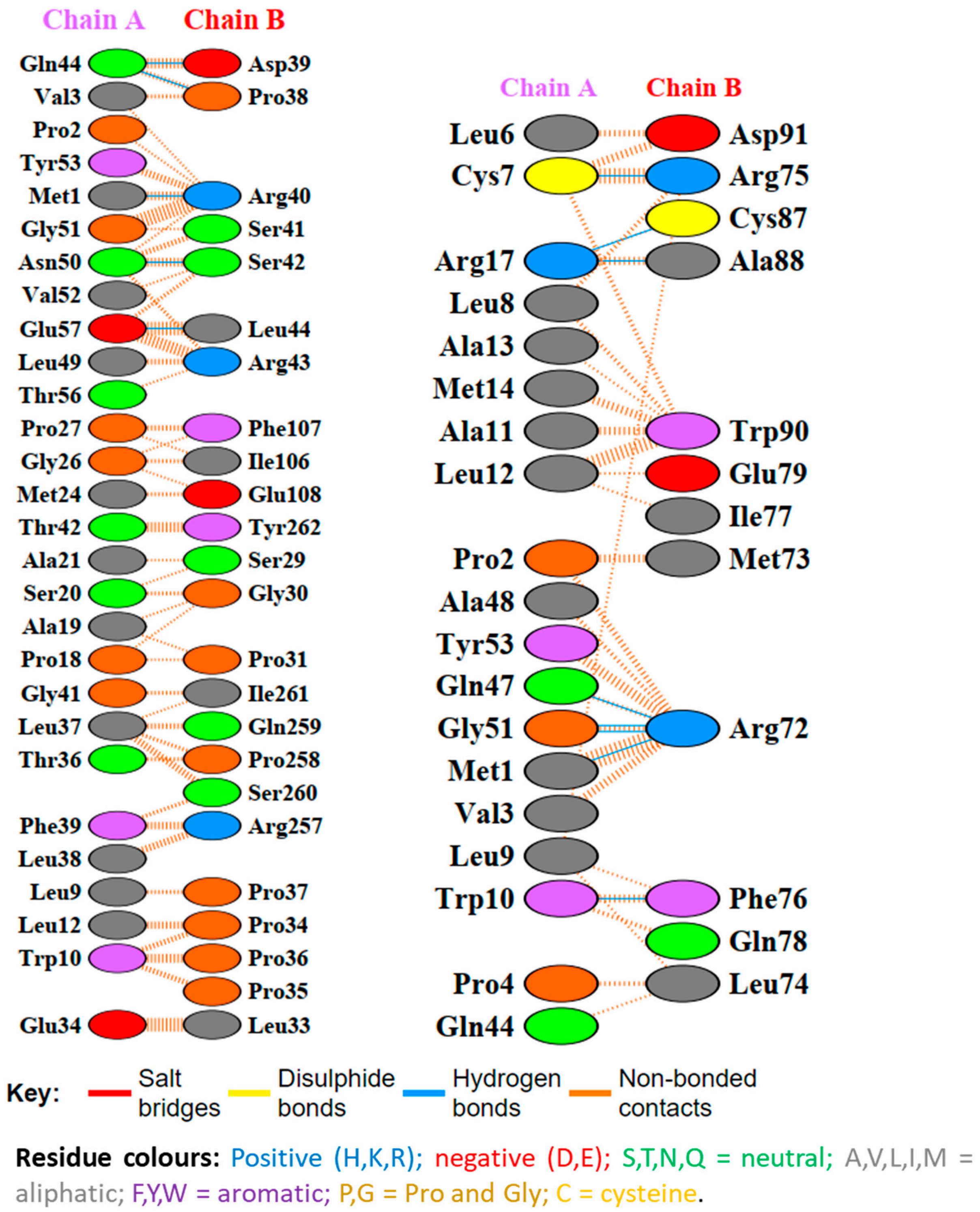

Figure S5. Interaction of ANGPTL8 (Chain A) with RASSF5 (Left) and FNDC5 (right).

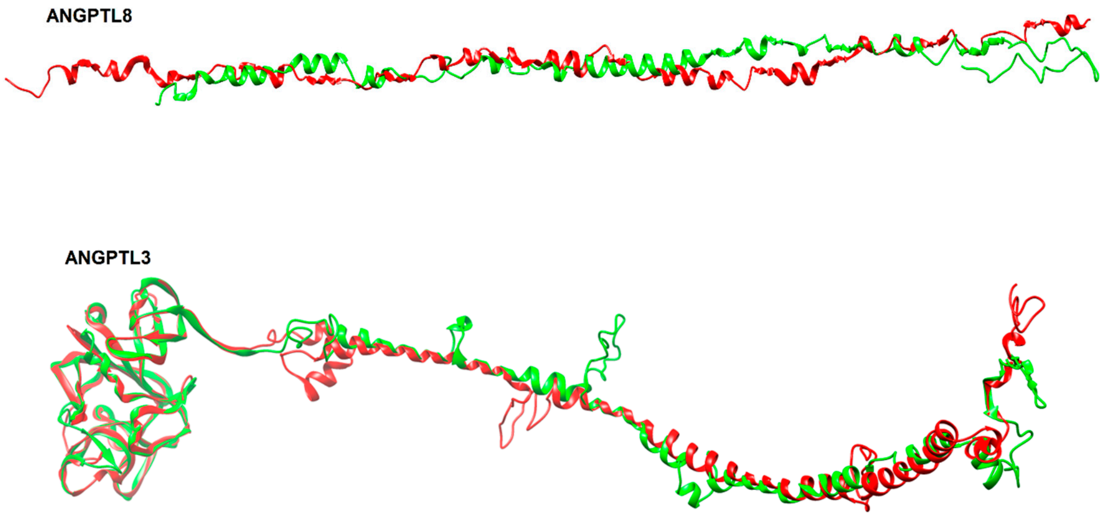

Figure S6. Comparative homology modeling of wild type and mutated ANGPTL3 and ANGPTL8. The wilt-type structures are shown in green while mutants are shown in red color.

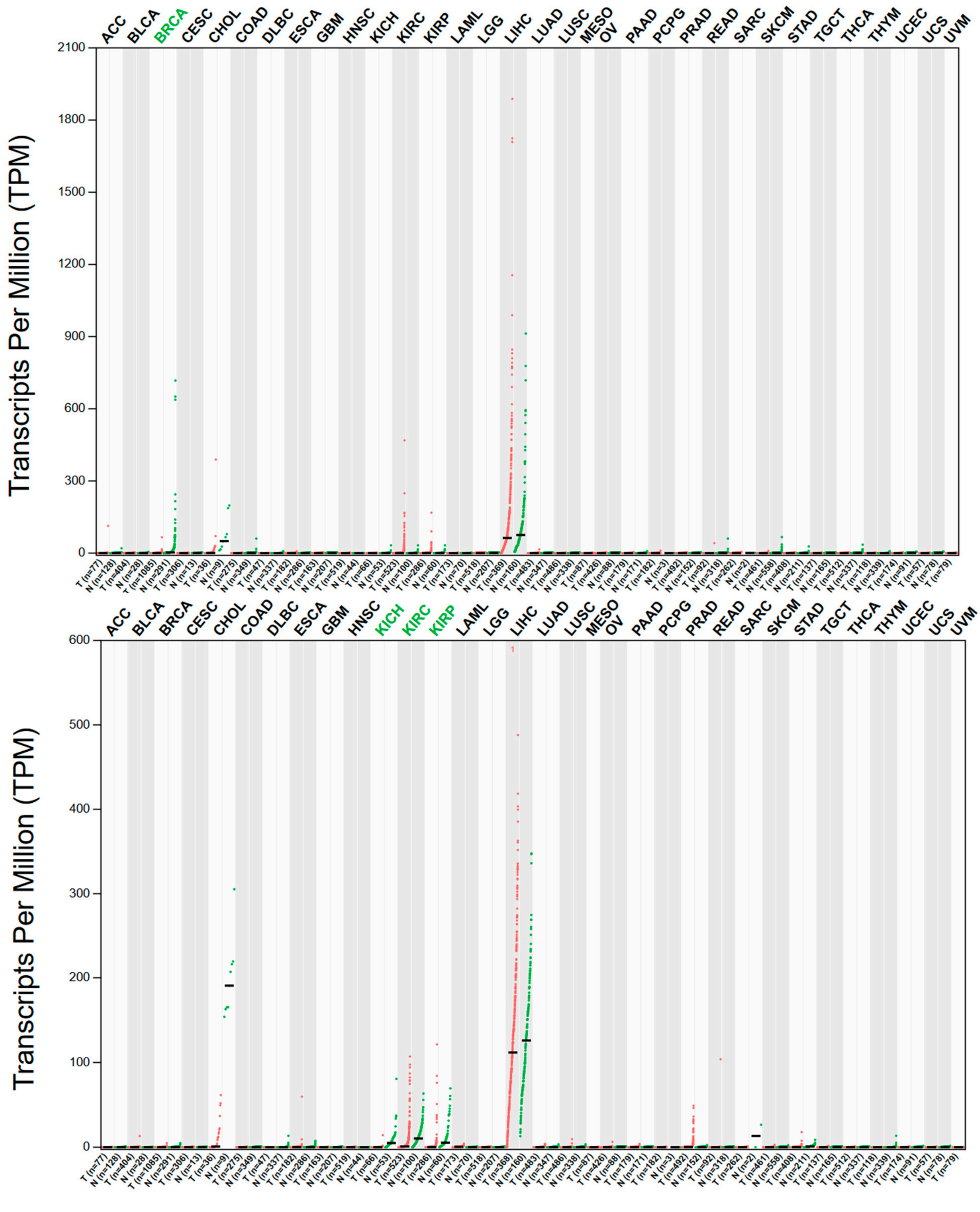

Figure S7. Gene expression analysis of *ANGPTL3* and *C19orf80* in all tumour types paired with normal tissue using the GEPIA database.
