# Supplementary material for: An Integrated Computational Analysis of High-Risk SNPs in Angiopoietin-like Proteins (ANGPTL3 and ANGPTL8) Reveals Perturbed Protein Dynamics Associated with Cancer
Source: Molecules. 2023 Jun 8;28(12):4648. doi: 10.3390/molecules28124648 (PMC10303706; doi:10.3390/molecules28124648)
Supplement: Supplementary file 1 [file molecules-28-04648-s001.zip › Supplementary file S3.pdf]

Table S1. HOPE analysis showed the consequences of nsSNPs in ANGPTL3.

**WT Amino acid**	**Position**	**M Amino acid**	**Properties and possible consequences**
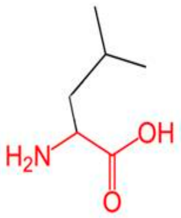 Leucine	6	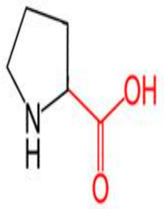 Proline	The M residue is smaller WT, this might lead to loss of external interactions.<br><br>The mutation is located within the signal peptide. This sequence of the peptide is important because it is recognized by other proteins and often cleaved off to generate the mature protein. The M residue that is introduced in the signal peptide differs in its properties from the WT. This mutation may disturb recognition of the signal peptide.
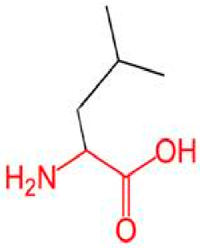 Leucine	50	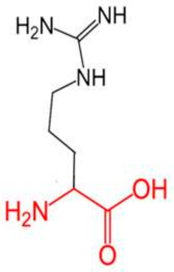 Arginine	There is a difference in charge between the WT and mutant amino acid.<br><br>The mutation introduces a charge, this can cause repulsion of ligands or other residues with the same charge.<br><br>The mutant residue is bigger, this might lead to bumps.<br><br>The hydrophobicity of the wild-type and mutant residue differs. Hydrophobic interactions, either in the core of the protein or on the surface, will be lost.
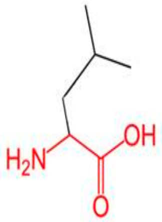 Leucine	53	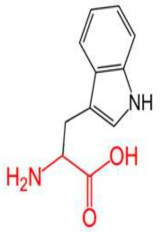 Tryptophan	The WT and mutant amino acids differ in size. The M residue is bigger, this might lead to bumps.
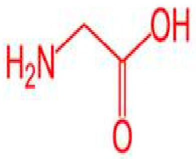 Glycine	54	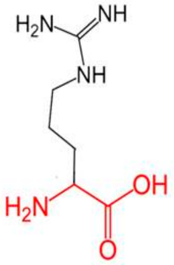 Arginine	The WT residue is a glycine, the most flexible of all residues. This flexibility might be necessary for the protein's function. Mutation of this glycine can abolish this function.<br><br>The WT amino acid is neutral and M is positively charged this can cause repulsion of ligands or other residues with the same charge.<br><br>The mutant residue is bigger, this might lead to bumps.<br><br>The torsion angles for this residue are unusual. Only glycine is flexible enough to make these torsion angles, mutation into another residue will force the local backbone into an incorrect conformation and will disturb the local structure.

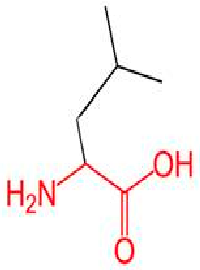 Leucine	57	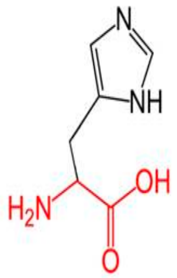 Histidine	The M residue is bigger, this might lead to bumps.<br><br>The hydrophobicity of the WT and M residue differs. Hydrophobic interactions, either in the core of the protein or on the surface, will be lost.<br><br>The mutation is located within a stretch of residues annotated in UniProt as a special region: Sufficient to inhibit LIPG/EL phospholipase activity. The differences in amino acid properties can disturb this region and disturb its function.
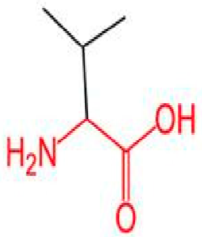 Valine	61	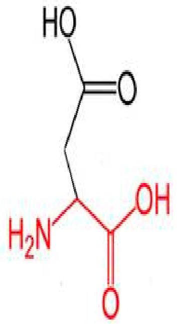 Aspartic Acid	The mutation is located within a stretch of residues annotated in UniProt as a special region: Sufficient to inhibit LIPG/EL phospholipase activity. The differences in amino acid properties can disturb this region and disturb its function.<br>The mutation introduces a charge, this can cause repulsion of ligands or other residues with the same charge.<br><br>The mutant residue is bigger, this might lead to bumps.<br><br>The hydrophobicity of the wild-type and mutant residue differs. Hydrophobic interactions, either in the core of the protein or on the surface, will be lost.
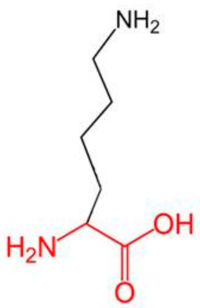 Lysine	63	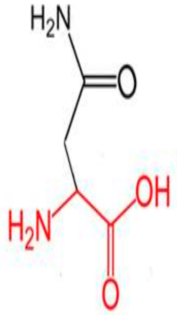 Asparagine	There is a difference in charge between the WT and mutant amino acid. The charge of the WT residue will be lost, this can cause loss of interactions with other molecules or residues.<br><br>The M residue is smaller, this might lead to loss of interactions.<br><br>The mutation is located within a stretch of residues annotated in UniProt as a special region: Sufficient to inhibit LIPG/EL phospholipase activity. The differences in amino acid properties can disturb this region and disturb its function.
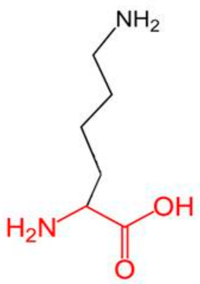 Lysine	63	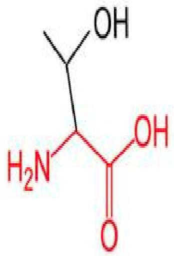 Threonine	There is a difference in charge between the wild-type and mutant amino acid. The charge of the wild-type residue will be lost, this can cause loss of interactions with other molecules or residues.<br><br>The mutant residue is smaller, this might lead to loss of interactions.<br><br>The hydrophobicity of the wild-type and mutant residue differs. The mutation introduces a more hydrophobic residue at this position. This can result in loss of hydrogen bonds and/or disturb correct folding.<br><br>Mutagenesis experiments have been performed on this position. Mutation of the WT residue into N has the following effect: Abolishes inhibitory effect on LIPG/EL phospholipase activity; when associated with N-65.

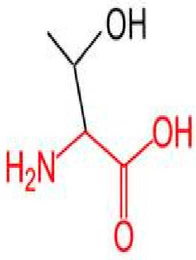 Threonine	64	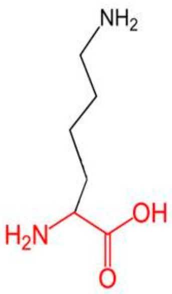 Lysine	The M amino acid is positively charged, this can cause repulsion of ligands or other residues with the same charge.<br><br>The M residue is bigger, this might lead to bumps.<br><br>The hydrophobicity of the wild-type and mutant residue differs. Hydrophobic interactions, either in the core of the protein or on the surface, will be lost.<br><br>The mutation is located in special stretch/region so, the differences in amino acid properties can disturb this region and disturb its function.
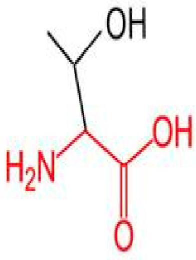 Threonine	64	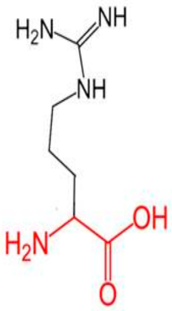 Arginine	The M amino acid carry positive charge this can cause repulsion of ligands or other residues with the same charge.<br><br>The mutant residue is bigger, this might lead to bumps.<br><br>The hydrophobicity of the wild-type and mutant residue differs. Hydrophobic interactions, either in the core of the protein or on the surface, will be lost.
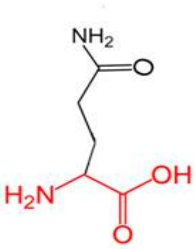 Glutamine	67	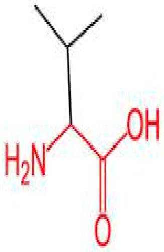 Valine	The mutant residue is smaller, this might lead to loss of interactions.<br><br>The hydrophobicity of the wild-type and mutant residue differs.<br><br>The mutation introduces a more hydrophobic residue at this position. This can result in loss of hydrogen bonds and/or disturb correct folding.
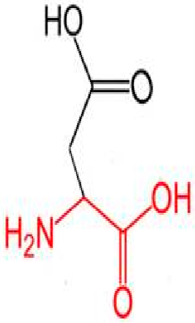 Aspartic Acid	79	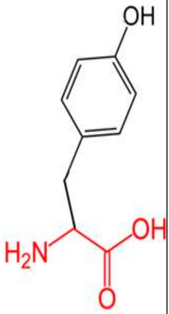 Tyrosine	There is a difference in charge between the WT and M amino acid. The charge of the wild-type residue will be lost, this can cause loss of interactions with other molecules or residues.<br><br>The M residue is bigger, this might lead to bumps.<br><br>The hydrophobicity of the WT and M residue differs. The mutation introduces a more hydrophobic residue at this position. This can result in loss of H-bonds and/or disturb correct folding.

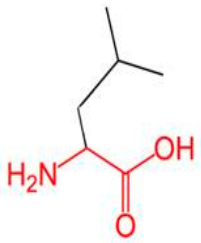 Leucine	87	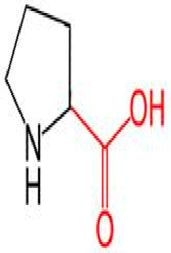 Proline	The mutant residue is smaller, this might lead to loss of interactions.<br><br>The mutation is located within a stretch of residues annotated in UniProt as a special region. The differences in amino acid properties can disturb this region and disturb its function.
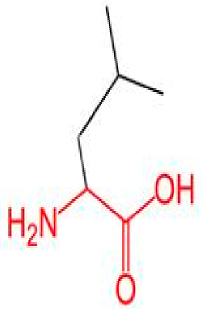 Leucine	124	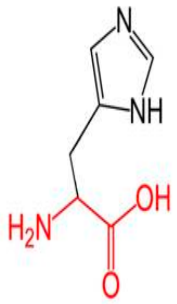 Histidine	The M residue is bigger, this might lead to bumps.<br><br>The hydrophobicity of the WT and mutant residue differs. Hydrophobic interactions, either in the core of the protein or on the surface, will be lost.<br><br>The mutation is located within a stretch of residues annotated in UniProt as a special region. The differences in amino acid properties can disturb this region and disturb its function.
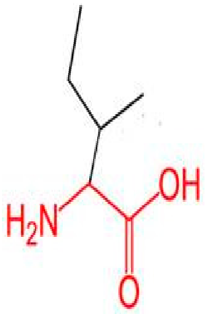 Isoleucine	175	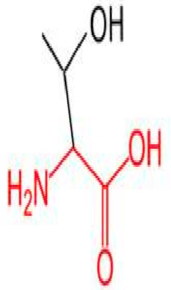 Threonine	The M residue is smaller than WT, this might lead to loss of interactions.<br><br>The hydrophobicity of the wild-type and mutant residue differs. Hydrophobic interactions, either in the core of the protein or on the surface, will be lost.<br><br>The mutation is located within special region. The differences in amino acid properties can disturb this region and disturb its function.
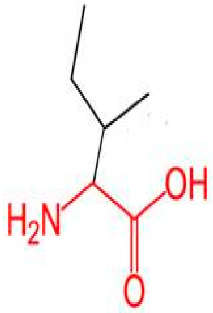 Isoleucine	196	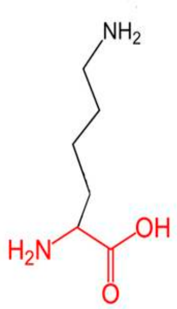 Lysine	The mutation introduces a charge, this can cause repulsion of ligands or other residues with the same charge.<br><br>The M residue is bigger, this might lead to bumps.<br><br>The hydrophobicity of the WT and M residue differs. Hydrophobic interactions, either in the core of the protein or on the surface, will be lost.

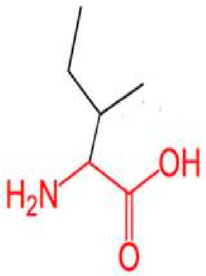<br><br>Isoleucine	196	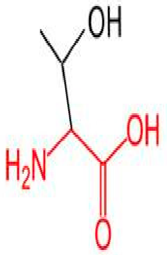<br><br>Threonine	The M residue is smaller, this might lead to loss of interactions.<br><br>The hydrophobicity of the WT and M residue differs. Hydrophobic interactions, either in the core of the protein or on the surface, will be lost. The mutation is located within a special stretch. The differences in amino acid properties can disturb this region and disturb its function.
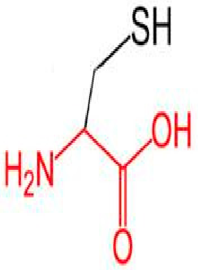<br><br>Cysteine	246	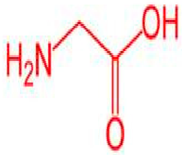<br><br>Glycine	The M residue is smaller than the WT residue. The mutation will cause an empty space in the core of the protein.<br><br>The hydrophobicity of the WT and mutant residue differs. The mutation will cause loss of hydrophobic interactions in the core of the protein.<br><br>The WT residue is annotated in UniProt to be involved in a cysteine bridge, which is important for stability of the protein. The mutation causes loss of this interaction and will have a severe effect on the 3D-structure of the protein.
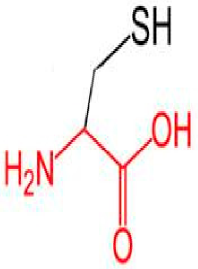<br><br>Cysteine	246	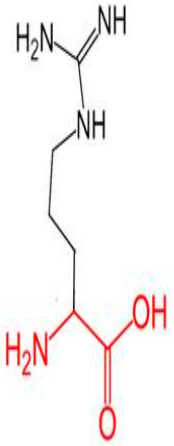<br><br>Arginine	There is a difference in charge between the WT and M amino acid.<br><br>The M residue introduces a charge in a buried residue which can lead to protein folding problems.<br><br>The WT residue was buried in the core of the protein. The mutant residue is bigger and probably will not fit.<br><br>The hydrophobicity of the WT and M residue differs. The mutation will cause loss of hydrophobic interactions in the core of the protein.<br><br>Cysteine make bridge with other amino acid, which is important for stability of the protein. Mutation in cysteine causes loss of this interaction and will have a severe effect on protein structure and function.
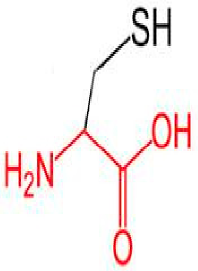<br><br>Cysteine	246	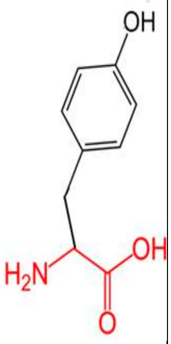<br><br>Tyrosine	The M residue is bigger than the WT residue and buried in the core of the protein. The mutant residue is bigger and probably will not fit.<br><br>The hydrophobicity of the WT and M residue differs that will cause loss of hydrophobic interactions in the core of the protein.<br><br>The WT residue is annotated in UniProt to be involved in a cysteine bridge, which is important for stability of the protein. The mutation causes loss of this interaction and will have a severe effect on the 3D-structure and function of the protein.

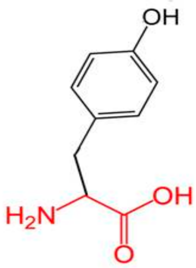<br>Tyrosine	250	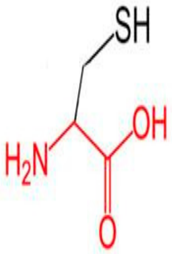<br>Cysteine	The M residue is smaller and less hydrophobic than the WT residue. This will cause a possible loss of external interactions.
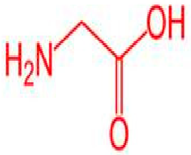<br>Glycine	253	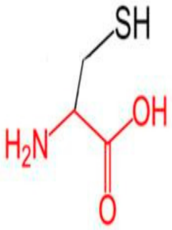<br>Cysteine	The M residue is bigger than the WT residue and located on the surface of the protein, mutation of this residue can disturb interactions with other molecules or other parts of the protein.<br>The torsion angles for this residue are unusual. only glycine is flexible enough to make these torsion angles, mutation into another residue will force the local backbone into an incorrect conformation and will disturb the local structure.
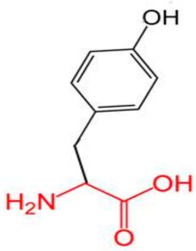<br>Tyrosine	260	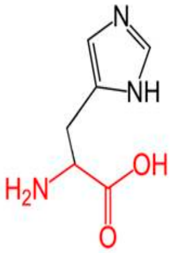<br>Histidine	The M residue is smaller, this might lead to loss of interactions.<br>The hydrophobicity of the WT and M residue differs. Hydrophobic interactions, either in the core of the protein or on the surface, will be lost.
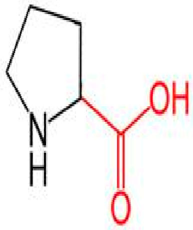<br>Proline	264	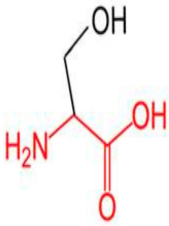<br>Serine	The M residue is smaller than the WT residue. The mutation will cause an empty space in the core of the protein.<br>The WT residue is more hydrophobic than the M residue.The mutation will cause loss of hydrophobic interactions in the core of the protein.
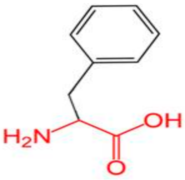<br>Phenylalanine	270	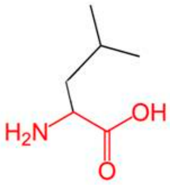<br>Leucine	The M residue is smaller than the WT residue.<br>The mutation will cause an empty space in the core of the protein.

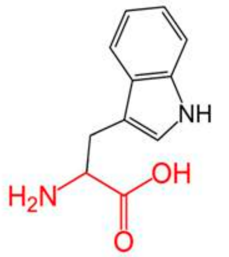 Tryptophan	282	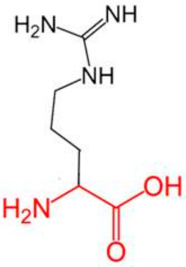 Arginine	The WT residue charge was Neutral, the mutant residue charge is POSITIVE. The mutation introduces a charge at this position, this can cause repulsion between the mutant residue and neighboring residues.<br>The Mt residue is smaller than the WT residue. This will cause a possible loss of external interactions.<br>The WT residue is more hydrophobic than the mutant residue. The mutation might cause loss of hydrophobic interactions with other molecules on the surface of the protein.
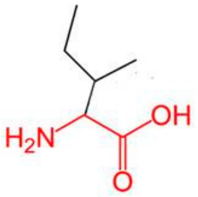 Isoleucine	285	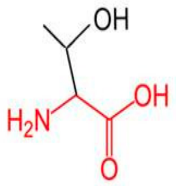 Threonine	The M residue is smaller than the WT residue. The mutation will cause an empty space in the core of the protein.<br>The WT residue is more hydrophobic than the M residue. The mutation will cause loss of hydrophobic interactions in the core of the protein.
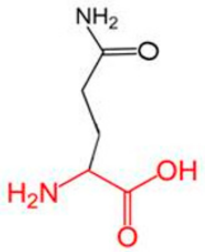 Glutamine	286	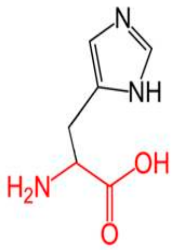 Histidine	The WT residue is smaller than the M residue and was buried in the core of the protein. The mutant residue is bigger and probably will not fit.<br>The WT residue forms H-bond with Asparagine at position 414. The size difference between WT and M residue makes that the new residue is not in the correct position to make the same hydrogen bond as the original WT residue did.
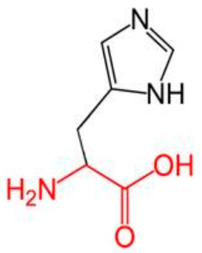 Histidine	287	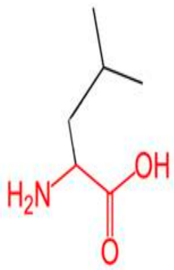 Leucine	The M residue is smaller than the WT residue. The mutation will cause an empty space in the core of the protein.<br>The hydrophobicity of the WT and M residue differs. The mutation will cause loss of hydrogen bonds in the core of the protein and as a result disturb correct folding.<br>The WT residue forms a H-bond with Aspartic Acid at position 310. The size difference between WT and M residue makes that the new residue is not in the correct position to make the same H-bond as the original WT residue did.
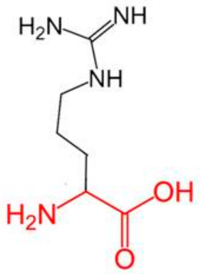 Arginine	288	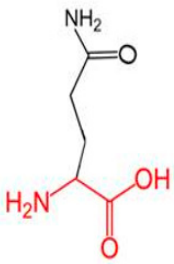 Glutamine	The WT residue forms a salt bridge with Aspartic Acid at position 290. The WT residue charge was POSITIVE, the M residue charge is NEUTRAL. The difference in charge will disturb the ionic interaction made by the original, WT residue.<br>The M residue is smaller than the WT residue. The mutation will cause an empty space in the core of the protein.

			The mutation is located in a region with known splice variants which abolishes protein secretion; associated with low plasma triglyceride level.
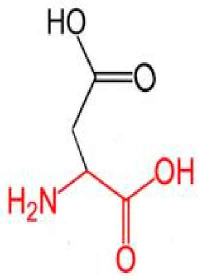 Aspartic Acid	290	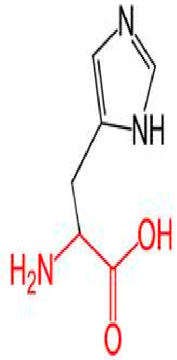 Histidine	The WT residue forms H- bond with Serine at position 292 and salt bridge with Arginine at position 288 and 308. The size and charge difference between WT and M residue makes that the new residue is not in the correct position to make the same H-bond and ionic interaction made by the WT residue.<br><br>The M residue is bigger than the WT residue. The residue is located on the surface of the protein, mutation of this residue can disturb interactions with other molecules or other parts of the protein.
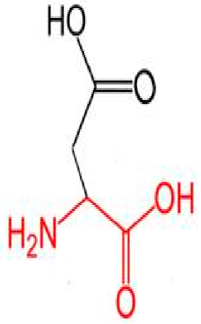 Aspartic Acid	290	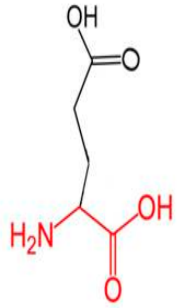 Glutamic Acid	The M residue is bigger than the WT residue. The residue is located on the surface of the protein, mutation of this residue can disturb interactions with other molecules or other parts of the protein.<br><br>The WT residue forms a H-bond with Serine at position 292 and salt bridge with Arginine at position 288 and 308. The size difference between WT and M residue makes that the new residue is not in the correct position to make the same H-bond as the WT residue did.
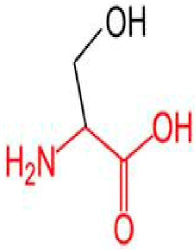 Serine	292	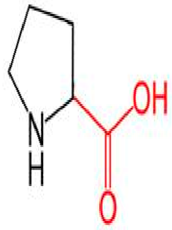 Proline	The M residue is bigger than the WT residue.<br><br>The WT residue forms a H-bond with Aspartic Acid at position 290. The hydrophobicity of the WT and M residue differs that will effect H-bond formation.<br><br>The residue is located on the surface of the protein, mutation of this residue can disturb interactions with other molecules or other parts of the protein.<br><br>The mutation is located in a region with known splice variants that abolishes protein secretion; associated with low plasma triglyceride level.
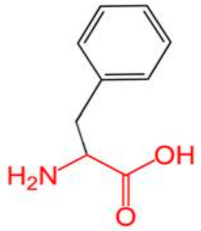 Phenylalanine	295	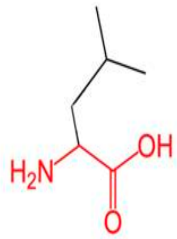 Leucine	The mutant residue is smaller than the wild-type residue. The mutation will cause an empty space in the core of the protein.<br><br>Only this residue type was found at this position. Mutation of a 100% conserved residue is usually damaging for the protein.

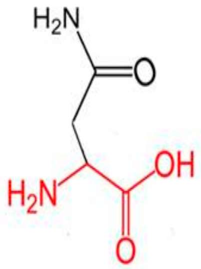 Asparagine	296	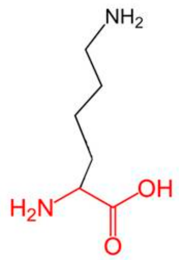 Lysine	The mutation introduces a charge at this position, this can cause repulsion between the mutant residue and neighboring residues.<br><br>The M residue is bigger than the WT residue and is located on the surface of the protein, mutation of this residue can disturb interactions with other molecules or other parts of the protein.
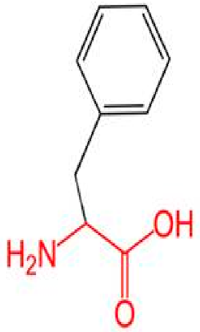 Phenylalanine	306	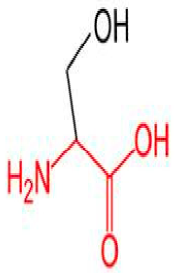 Serine	The M residue is smaller than the WT residue. This will cause a possible loss of external interactions.<br><br>Due to the difference in hydrophobicity of the WT and Mt residue the mutation might cause loss of hydrophobic interactions with other molecules on the surface of the protein. Only this residue type was found at this position. Mutation of a 100% conserved residue is usually damaging for the protein.
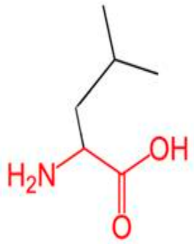 Leucine	309	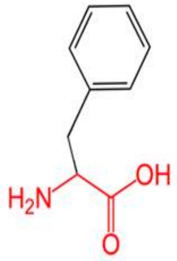 Phenylalanine	The M residue is bigger than the WT residue.<br><br>The residue is located on the surface of the protein, mutation of this residue can disturb interactions with other molecules or other parts of the protein.
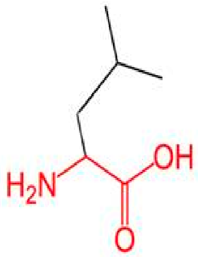 Leucine	315	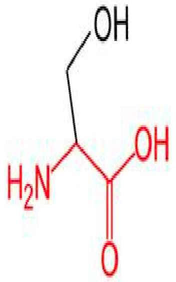 Serine	The M residue is smaller than the WT residue. The mutation will cause an empty space in the core of the protein.<br><br>The hydrophobicity of the WT and M residue differs. The mutation will cause loss of hydrophobic interactions in the core of the protein. Only this residue type was found at this position. Mutation of a 100% conserved residue is usually damaging for the protein.

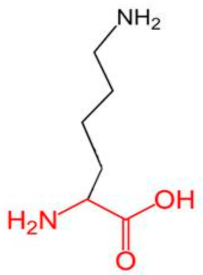 Lysine	319	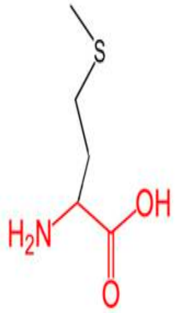 Methionine	There is a difference in charge between the WT and M amino acid. The charge of the WT residue is lost by this mutation. This can cause loss of interactions with other molecules.<br><br>The M residue is smaller than the WT residue. The hydrophobicity of the wild-type and mutant residue differs. This will cause a possible loss of external interactions.
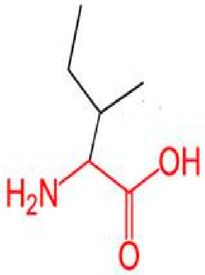 Isoleucine	320	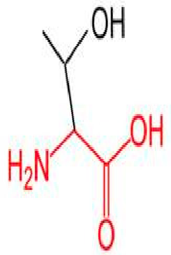 Threonine	The M residue is smaller than the WT residue. The mutation will cause an empty space in the core of the protein.<br><br>The hydrophobicity of the WT and M residue differs. The mutation will cause loss of hydrophobic interactions in the core of the protein.
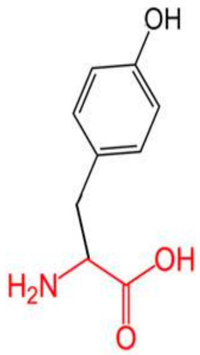 Tyrosine	321	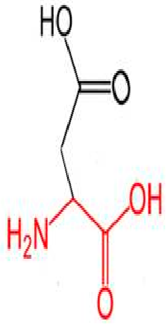 Aspartic Acid	The mutation introduces a charge at this position, this can cause repulsion between the mutant residue and neighboring residues.<br><br>The M residue is smaller than the WT residue. This will cause a possible loss of external interactions.<br><br>The hydrophobicity of the WT and M residue differs. The mutation might cause loss of hydrophobic interactions with other molecules on the surface of the protein.<br><br>The WT residue forms H-bond with Lysine at position 325. The size difference and hydrophobicity between WT and M residue makes that the new residue is not in the correct position to make the same H- bond as the WT residue did.
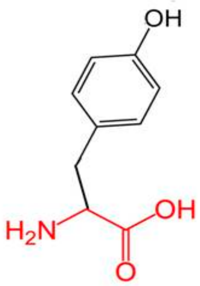 Tyrosine	329	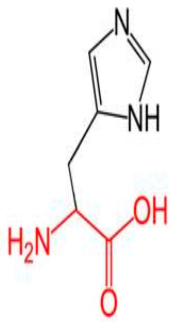 Histidine	The WT residue forms a H-bond with Glutamic Acid at position 245<br><br>The size and hydrophobicity difference between WT and M residue makes that the new residue is not in the correct position to make the same H-bond as the WT residue did.<br><br>The M residue is smaller than the WT residue. That will cause an empty space in the core of the protein. The mutation will cause loss of hydrophobic interactions in the core of the protein.

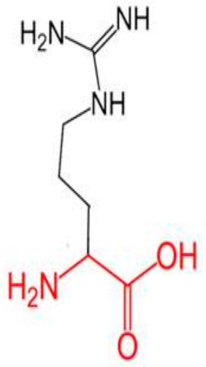 Arginine	332	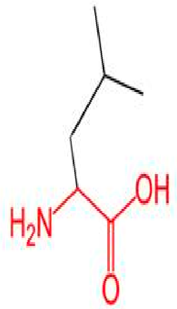 Leucine	The charge of the WT residue is lost by this mutation. This can cause loss of interactions with<br><br>The M residue is smaller than the WT residue. This will cause a possible loss of external interactions.<br><br>The WT residue forms a H-bond with Glutamic Acid at position 334. The size and hydrophobicity difference between WT and M residue makes that the new residue is not in the correct position to make the same H-bond as the original WT residue did.<br><br>The WT residue forms a salt bridge with Aspartic Acid at position 275 Glutamic Acid at position 334 and 346. The difference in charge will disturb the ionic interaction made by the WT residue.
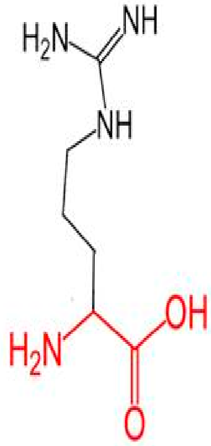 Arginine	332	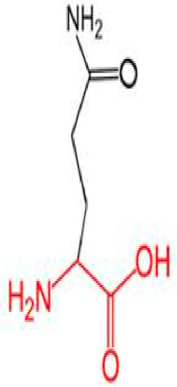 Glutamine	The charge of the WT residue is lost by this mutation. This can cause loss of interactions with other molecules.<br><br>The M residue is smaller than the WT residue. This will cause a possible loss of external interactions.<br><br>The WT residue forms H-bond with Glutamic Acid at position 334. The size difference between WT and mutant residue makes that the new residue is not in the correct position to make the same H-bond as the original WT residue did.<br><br>The WT residue forms a salt bridge with Aspartic Acid at position 275, Glutamic Acid at position 334 and 346. The difference in charge will disturb the ionic interaction made by the WT residue.
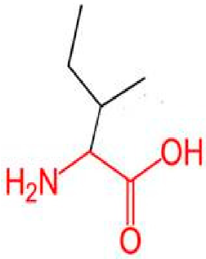 Isoleucine	333	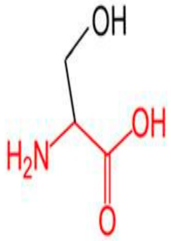 Serine	The M residue is smaller than the WT residue. The mutation will cause an empty space in the core of the protein.<br><br>The hydrophobicity of the wild-type and mutant residue differs. The mutation will cause loss of hydrophobic interactions in the core of the protein.
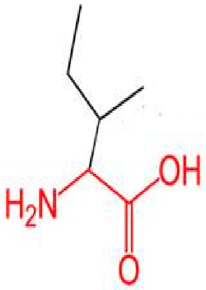 Isoleucine	333	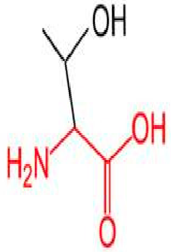 Threonine	The M residue is smaller than the WT residue. The mutation will cause an empty space in the core of the protein.<br><br>The hydrophobicity of the wild-type and mutant residue differs. The mutation will cause loss of hydrophobic interactions in the core of the protein.

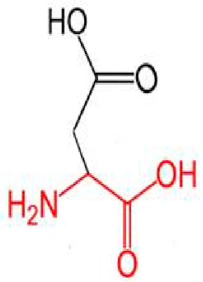 Aspartic Acid	337	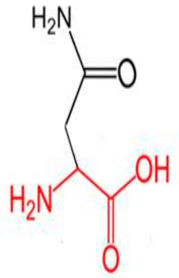 Asparagine	There is a difference in charge between the WT and M amino acid. The charge of the buried WT residue is lost by this mutation.<br><br>The WT residue forms a salt bridge with Lysine at position 339 and with Arginine at position 421. The difference in charge will disturb the ionic interaction made by the WT residue.<br><br>Only this residue type was found at this position. Mutation of a 100% conserved residue is usually damaging for the protein.
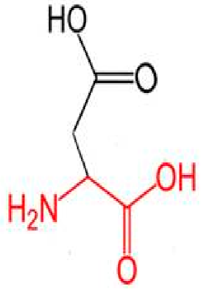 Aspartic	337	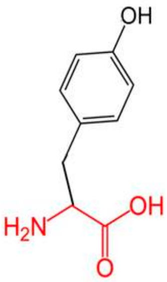 Tyrosine	The WT residue forms a salt bridge with Lysine at position 339 and with Arginine at position 421. The difference in charge will disturb the ionic interaction made by the WT residue.<br><br>The WT residue was buried in the core of the protein. The mutant residue is bigger and probably will not fit.<br><br>Due to the difference in hydrophobicity this mutation will cause loss of H-bonds in the core of the protein and as a result disturb correct folding.<br><br>Only this residue was found at this position. Mutation of a 100% conserved residue is usually damaging for the protein.
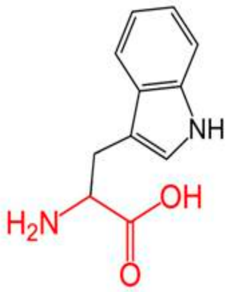 Tryptophan	338	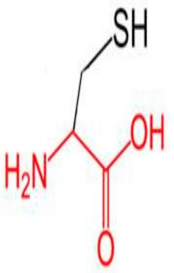 Cysteine	The mutant residue is smaller than the wild-type residue. This will cause a possible loss of external interactions.
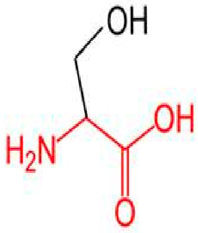 Serine	348	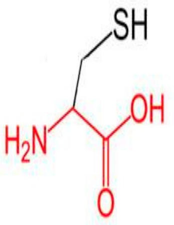 Cysteine	The WT residue forms H-bond with Alanine at position 364<br><br>The hydrophobicity of the WT and M residue differs will affect H-bond formation as a result disturb correct folding.

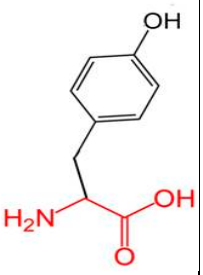 Tyrosine	358	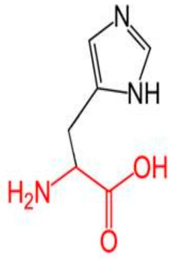 Histidine	The M residue is smaller than the WT residue. The mutation will cause an empty space in the core of the protein.<br>The hydrophobicity of the wild-type and mutant residue differs. This will cause loss of hydrophobic interactions in the core of the protein.
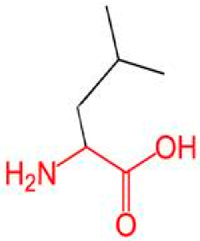 Leucine	360	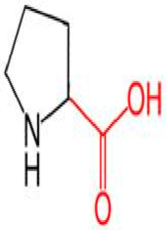 Proline	The M residue is smaller than the WT residue. This will cause a possible loss of external interactions.
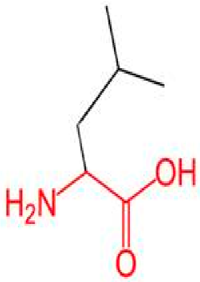 Leucine	360	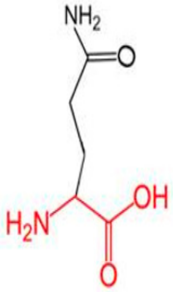 Glutamine	The M residue is bigger than the WT residue. The residue is located on the surface of the protein, mutation of this residue can disturb interactions with other molecules or other parts of the protein.<br>The hydrophobicity of the WT and M residue differs. Thus this mutation might cause loss of hydrophobic interactions with other molecules on the surface of the protein.
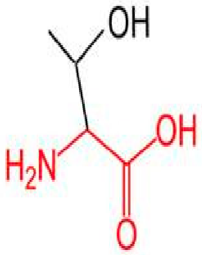 Threonine	383	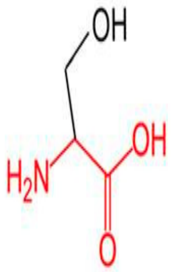 Serine	The WT residue forms a H-bond with Serine at position 382. The size difference between WT and M residue makes that the new residue is not in the correct position to make the same H-bond as the original WT residue did.<br>The M residue is smaller than the WT residue. The mutation will cause an empty space in the core of the protein.
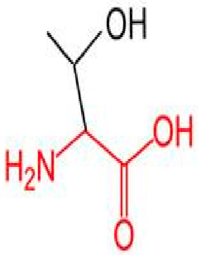 Threonine	383	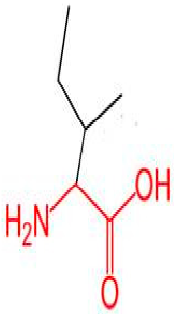 Isoleucine	The M residue is bigger than the WT residue. The wild-type residue was buried in the core of the protein. The mutant residue is bigger and probably will not fit.<br>The hydrophobicity of the wild-type and mutant residue differs. The mutation will cause loss of hydrogen bonds in the core of the protein and as a result disturb correct folding.<br>The WT residue forms a H-bond with Serine at position 382. The size difference between WT and mutant residue makes that the new residue is not in the correct

			position to make the same hydrogen bond as the original wild-type residue did.<br><br>The difference in hydrophobicity will affect H-bond formation.
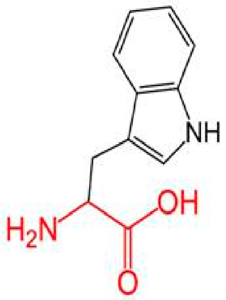<br>Tryptophan	384	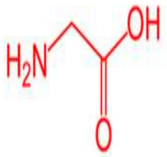<br>Glycine	The M residue is smaller than the WT residue. This will cause a possible loss of external interactions.<br><br>The hydrophobicity of the WT and M residue differs. The mutation might cause loss of hydrophobic interactions with other molecules on the surface of the protein.<br><br>The mutated residue is not in contact with a metal, however, one of the neighbouring residues does make a metal-contact that might be affected by the mutation in its vicinity.
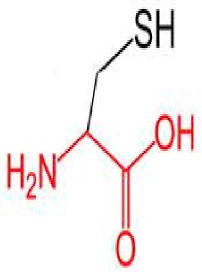<br>Cysteine	394	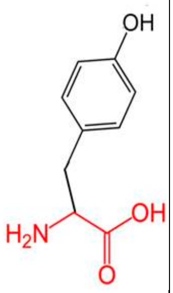<br>Tyrosine	The WT residue is smaller and was buried in the core of the protein. The M residue is bigger and probably will not fit.<br><br>The hydrophobicity of the WT and M residue differs. The mutation will cause loss of hydrophobic interactions in the core of the protein.<br><br>The WT residue is involved in a cysteine bridge, which is important for stability of the protein. Only cysteines can make these type of bonds, the mutation causes loss of this interaction and will have a severe effect on the 3D-structure of the protein.
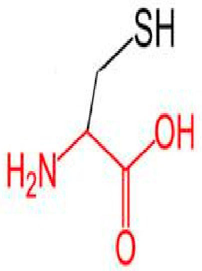<br>Cysteine	408	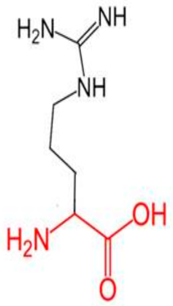<br>Arginine	There is a difference in charge between the WT and M amino acid. The M amino acid is positively charged, this can cause repulsion between the mutant residue and neighboring residues.<br><br>The M residue is bigger than the WT residue. The residue is located on the surface of the protein, mutation of this residue can disturb interactions with other molecules or other parts of the protein.<br><br>The hydrophobicity of the WT and M residue differs. That might cause loss of hydrophobic interactions with other molecules on the surface of the protein.
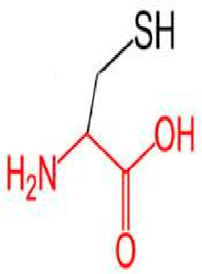<br>Cysteine	408	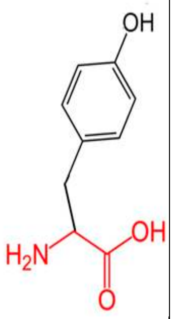<br>Tyrosine	The mutant residue is bigger than the wild-type residue.<br><br>The residue is located on the surface of the protein, mutation of this residue can disturb interactions with other molecules or other parts of the protein.<br><br>The hydrophobicity of the wild-type and mutant residue differs. The mutation might cause loss of hydrophobic interactions with other molecules on the surface of the protein.<br><br>The wild-type residue is annotated in UniProt to be involved in a cysteine bridge, which is important for stability of the protein. Only cysteines can make these

			type of bonds, the mutation causes loss of this interaction and will have a severe effect on the 3D-structure of the protein.
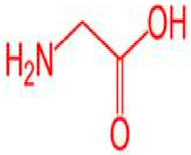<br>Glycine	409	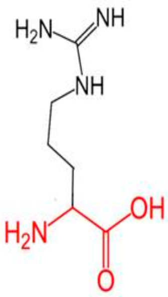<br>Arginine	The WT residue charge was neutral, the M residue charge is positive which can cause repulsion between the mutant residue and neighboring residues.<br><br>The M residue is bigger than the WT residue and is located on the surface of the protein, mutation of this residue can disturb interactions with other molecules or other parts of the protein.<br><br>Only glycine is flexible enough to make this unusual torsion angles, mutation into another residue will force the local backbone into an incorrect conformation and will disturb the local structure.
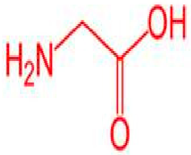<br>Glycine	415	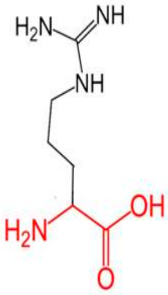<br>Arginine	The M residue introduces a charge in a buried residue which can lead to protein folding problems.<br><br>The M residue is bigger than the WT residue.<br><br>The WT residue was buried in the core of the protein. The M residue is bigger and probably will not fit.<br><br>The torsion angles for this residue are unusual. Only glycine is flexible enough to make these torsion angles, mutation into another residue will force the local backbone into an incorrect conformation and will disturb the local structure.
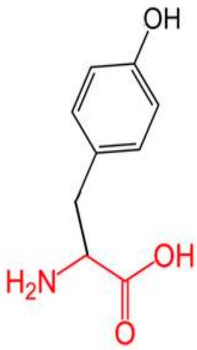<br>Tyrosine	417	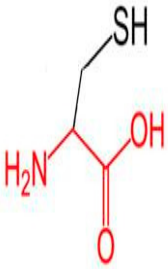<br>Cysteine	The WT residue forms a H-bond with Isoleucine at position 289. The size difference between WT and M residue makes that the new residue is not in the correct position to make the same H-bond as the original WT residue did.<br><br>The M residue is smaller than the WT residue. This will cause a possible loss of external interactions.<br><br>The mutation is located in a region with known splice variants, described as: The severity polymorphism, Abolishes protein secretion associated with low plasma triglyceride level.
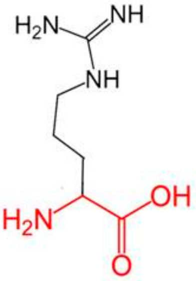<br>Arginine	428	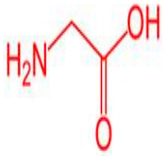<br>Glycine	The WT residue charge was positive, the mutant residue charge is neutral. This can cause loss of interactions with other molecules.<br><br>The M residue is smaller than the WT residue. This will cause a possible loss of external interactions.

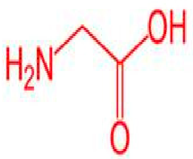<br>Glycine	439	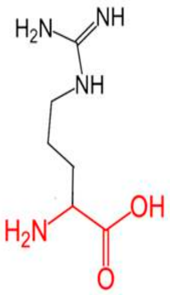<br>Arginine	The WT residue charge was neutral, the M residue charge is positive. this can cause repulsion between the mutant residue and neighboring residues.<br>The M residue is bigger than the WT residue.<br>The residue is more hydrophbic and located on the surface of the protein, mutation of this residue can disturb interactions with other molecules or other parts of the protein.
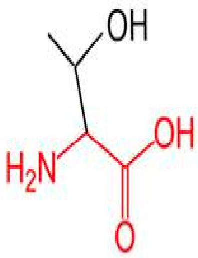<br>Threonine	447	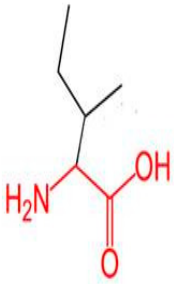<br>Isoleucine	The M residue is bigger than the WT residue.<br>The WT amino acid is smaller than M and was buried in the core of the protein. So, the M residue probably will not fit.<br>The hydrophobicity of the WT and M residue differs. The mutation will cause loss of hydrogen bonds in the core of the protein and as a result disturb correct folding.
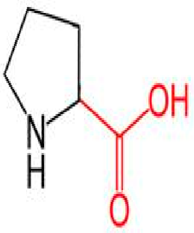<br>Proline	453	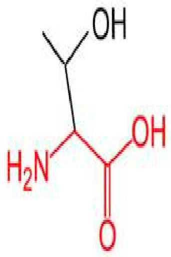<br>Threonine	The WT residue is more hydrophobic than the M residue. The mutation might cause loss of hydrophobic interactions with other molecules on the surface of the protein.

Table S2. HOPE analysis for putative consequences of nsSNPs in ANGPTL 8

WT Residue	Position	Mutant Residue	Description
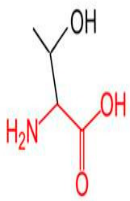<br>Threonine	56	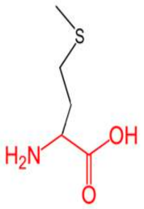<br>Methionine	The mutant residue is bigger, this might lead to bumps.<br>The mutation introduces a more hydrophobic residue at this position. This can result in loss of hydrogen bonds and/or disturb correct folding.

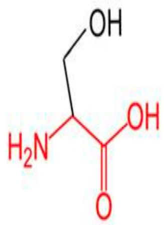<br>Serine	66	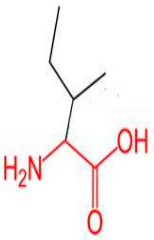<br>Isoleucine	The mutant residue is bigger, this might lead to bumps.<br>The hydrophobicity of the wild-type and mutant residue differs. The mutation introduces a more hydrophobic residue at this position. This can result in loss of hydrogen bonds and/or disturb correct folding.
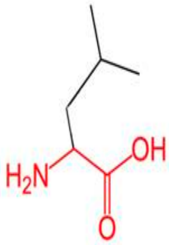<br>Leucine	77	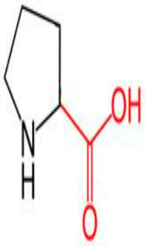<br>Proline	The wild-type residue is predicted (using the Reprof software) to be located in an α-helix.<br>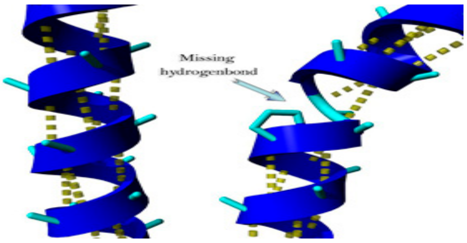<br><br>Proline disrupts an α-helix when not located at one of the first 3 positions of that helix. In case of the mutation at hand, the helix will be disturbed and this can have severe effects on the structure of the protein.<br>The mutant residue is smaller, this might lead to loss of interactions.
	85		
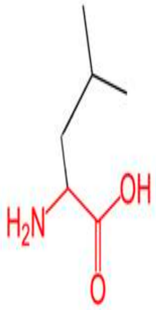<br>Leucine	133	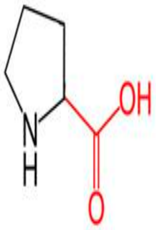<br>Proline	The wild-type residue is predicted to be located in an α-helix.<br>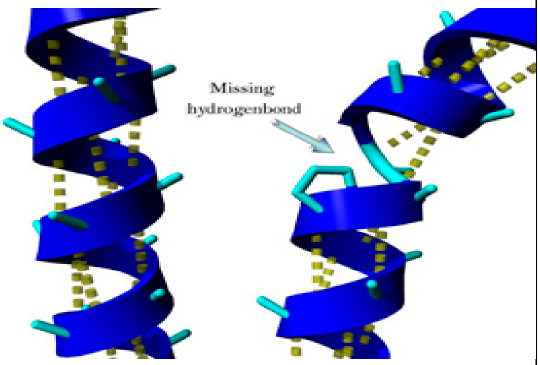<br><br>Proline disrupts an α-helix when not located at one of the first 3 positions of that helix. In case of the mutation at hand, the helix will be disturbed and this can have severe effects on the structure of the protein.

			Only this residue type was found at this position. Mutation of a 100% conserved residue is usually damaging for the protein.<br>The mutant residue is smaller than the wild-type residue. The mutation will cause an empty space in the core of the protein.
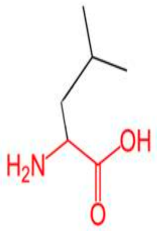<br>Leucine	137	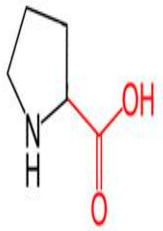<br>Proline	Only this residue type was found at this position. Mutation of a 100% conserved residue is usually damaging for the protein.<br>The mutant residue is smaller than the wild-type residue. This will cause a possible loss of external interactions.
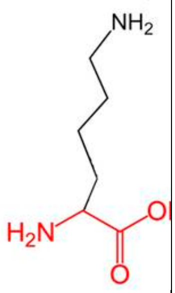<br>Lysine	153	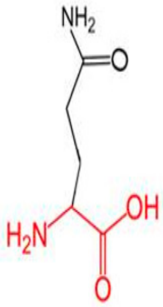<br>Glutamine	The wild-type residue forms a salt bridge with Glutamic Acid at position 110 and 150. The difference in charge will disturb the ionic interaction made by the original, wild-type residue.<br>In the 3D-structure can be seen that the wild-type residue is located in an α-helix. The mutation converts the wild-type residue in a residue that does not prefer α-helices as secondary structure.<br>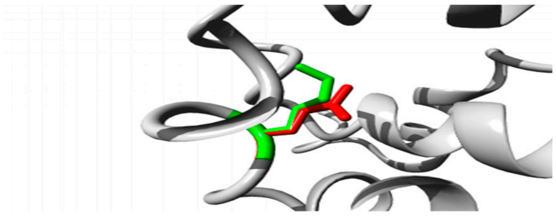<br>Only this residue type was found at this position. Mutation of a 100% conserved residue is usually damaging for the protein.<br>The mutant residue is smaller this will cause an empty space in the core of the protein.
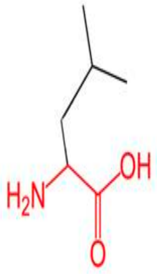	166	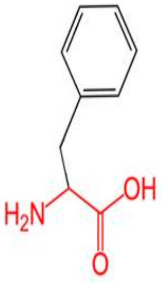	The wild-type residue is predicted to be located in an α-helix.<br>The mutation converts the wild-type residue in a residue that does not prefer α-helices as secondary structure.<br>The mutant residue is bigger, this might lead to bumps.

Leucine		Phenylalanine	
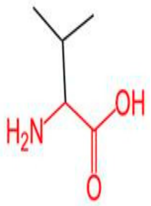 Valine	170	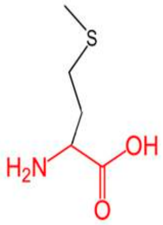 Methionine	The mutant residue is bigger, this might lead to bumps.
